# The non-linear and linear effects of CYP2C19 metaboliser status on DNA methylation: a methylome-wide association study

**DOI:** 10.1186/s13148-026-02125-w

**Published:** 2026-04-22

**Authors:** Chen Shen, Mark J. Adams, Eleanor Davyson, Matthew H. Iveson, Simon R. Cox, Sarah E. Harris, Andrew M. McIntosh, Xueyi Shen

**Affiliations:** 1 MRC Centre for Environment and Health, School of Public Health, Imperial College London, London, UK; 2 NIHR Health Protection Research Unit in Radiation Threats and Hazards, Imperial College London, London, UK; 3 Institute for Neuroscience and Cardiovascular Research, The University of Edinburgh, GU305, Chancellor’s Building, Royal Infirmary, 49 Little France Crescent, Edinburgh, EH16 4SB UK; 4 Lothian Birth Cohorts, Department of Psychology, The University of Edinburgh, Edinburgh, UK; 5 Lothian Birth Cohorts, Edinburgh Futures Institute, The University of Edinburgh, Edinburgh, UK

**Keywords:** CYP2C19, DNA methylation, Methylome-wide association study, Drug metabolism

## Abstract

**Background:**

CYP2C19 metabolises many medications. Its enzymatic activity can be inferred from genetic variants within *CYP2C19* that link to the efficacy of drug treatments and their side effects. It is, however, unclear if enzymatic activity is associated with local or widespread differences in DNA methylation. DNA methylation differences associated with CYP2C19 metabolising status may also reveal interacting genes and pathways that underlie CYP2C19 effects on drug response and health consequences. A discovery methylome-wide association study (MWAS) was conducted in the Generation Scotland (*n* = 18,396) to investigate the non-linear and linear effects of CYP2C19 metaboliser status on DNA methylation. A targeted replication analysis on significant CpG sites from the discovery MWAS was conducted in Lothian Birth Cohorts of 1921 and 1936 (*n* = 1238). Pathway enrichment analysis was conducted for significant cytosine-guanine dinucleotide (CpG) sites. We examined whether the associations between CYP2C19 metaboliser status and DNA methylation were independent of the use of drugs that are inducers, inhibitors, or substrates of the CYP2C19 enzyme through interaction analysis.

**Results:**

Forty-eight CpG sites were significantly associated with the quadratic term of CYP2C19 metaboliser status (P_Bonferroni_<0.05). Nineteen CpG sites, annotated to genes involving drug metabolism, inflammation, lipid levels, and Type 2 diabetes, demonstrated non-linear associations with CYP2C19 metaboliser status. Among the significant CpG sites in the discovery sample, there was a high correlation of standardised regression coefficients between the discovery and replication samples (*r* = 0.92). We found enrichment in biological processes involving metabolic activities and the Cytochrome P450 pathway. CYP2C19 metaboliser status did not interact with CYP2C19-related medication use to affect methylation of non-linear CpG signals.

**Conclusions:**

This research suggests that genetically-determined CYP2C19 metaboliser status is associated with both local and distal DNA methylation. These associations are independent of whether individuals were receiving drugs that are related to this enzyme.

**Supplementary Information:**

The online version contains supplementary material available at 10.1186/s13148-026-02125-w.

## Background

The activity of many drug-metabolising enzymes influences drug response and side effects. Therefore, drug metabolising status is likely to have an important role in precision medicine by guiding the selection and dosage of medications for patients. The cytochromes P450 (CYP) superfamily comprises multiple genes that code for enzymes involving the metabolism of various exogenous and endogenous compounds such as prescribed drugs, environmental pollutants, cholesterol, steroids, and Vitamin D [1]. The CYP family 2 subfamily C, polypeptide 19 (CYP2C19) enzyme, encoded by *CYP2C19* gene on chromosome 10 (10q24) [2], plays a key role in the metabolism of a wide range of clinically relevant drugs such as antidepressants, proton pump inhibitors (PPI), and antiplatelet medicines [3–5].

Genetic variants within the *CYP2C19* gene determine the metabolic activity of CYP2C19 enzyme, which has important implications for drug safety and efficacy. Recent clinical practices in the UK have begun considering CYP2C19 genotype testing to guide drug use (https://www.nice.org.uk/guidance/dg59). Some variants (e.g., CYP2C19*2, CYP2C19*3) are associated with reduced or loss of CYP2C19-mediated drug metabolising activity [6]. Carriers of these variants have lower levels of active metabolites converted by these drugs, resulting in reduced response to treatment and/or increased side effects. For instance, evidence has indicated a relationship between *CYP2C19* genetic variants associated with poor metabolism and intolerance of citalopram, accompanied by discontinuation of treatment due to side effects [7, 8]. Carriers of other *CYP2C19* variant alleles (e.g., CYP2C19*17, associated with ultrarapid metabolism) have increased drug metabolism capacity, which may result in a lack of response to standard antidepressant and PPI treatments due to an unusually rapid clearance of drugs [8, 9]. This may indicate a potential non-linear relationship between CYP2C19 metaboliser status and health implications with drug use. It has been found that both poor and ultrarapid CYP2C19 metabolisers are linked to a higher risk of therapeutic failure of escitalopram (an antidepressant) [10]. *CYP2C19* polymorphism is also related to the metabolism of wider environmental exposures such as toxicants (e.g., heavy metals, organic pollutants) and nutrients (e.g., omega-3 fatty acids) [11]. It has also been shown that individuals with high CYP2C19 enzymatic capacity (i.e., rapid/ultrarapid metaboliser) had more depressive symptoms regardless of antidepressant treatment [12, 13]. This indicates that the functional impact of CYP2C19 enzyme may extend beyond drug metabolism to broader physiological and psychiatric relevance.

Epigenetic change can act as an environmental archive of exogenous exposures (including medication use) by reflecting duration, intensity, and individual susceptibility to adverse environmental risk factors [14]. DNA methylation at cytosine-phosphate-guanine (CpG) sites is one of the most commonly investigated epigenetic processes. DNA methylation has established connections with environmental factors and provides important insights into the functional genomic mechanisms underlying common diseases [15]. Previous research suggests that expression of genes that encode drug metabolising enzymes (e.g., *CYP2C19*,* GSTA4*,* UGT1A1*) is influenced by DNA methylation. Such variations in gene expression may result in individual differences in drug response [16, 17]. However, these studies have only investigated methylation within specific candidate genes, limiting insight into the broader impact of drug metaboliser enzymatic activity on health. To our knowledge, no methylome-wide association studies (MWAS) have investigated the association between metaboliser status determined by *CYP2C19* variants and genome-wide DNA methylation. It is unclear if enzymatic activity is associated with local or widespread differences in DNA methylation, which may reveal interacting genes and pathways that underlie CYP2C19 effects on drug response and health consequences. Whilst linear trends can inform DNA methylation patterns in relation to CYP2C19 metaboliser status, it is possible that associations are driven by genetic determinants of DNA methylation (methylation quantitative trait loci (mQTL)). Investigation of both non-linear and linear relationships can help deepen biological insights into how CYP2C19 metaboliser status affects DNA methylation, where current evidence is limited.

**Fig. 1:**
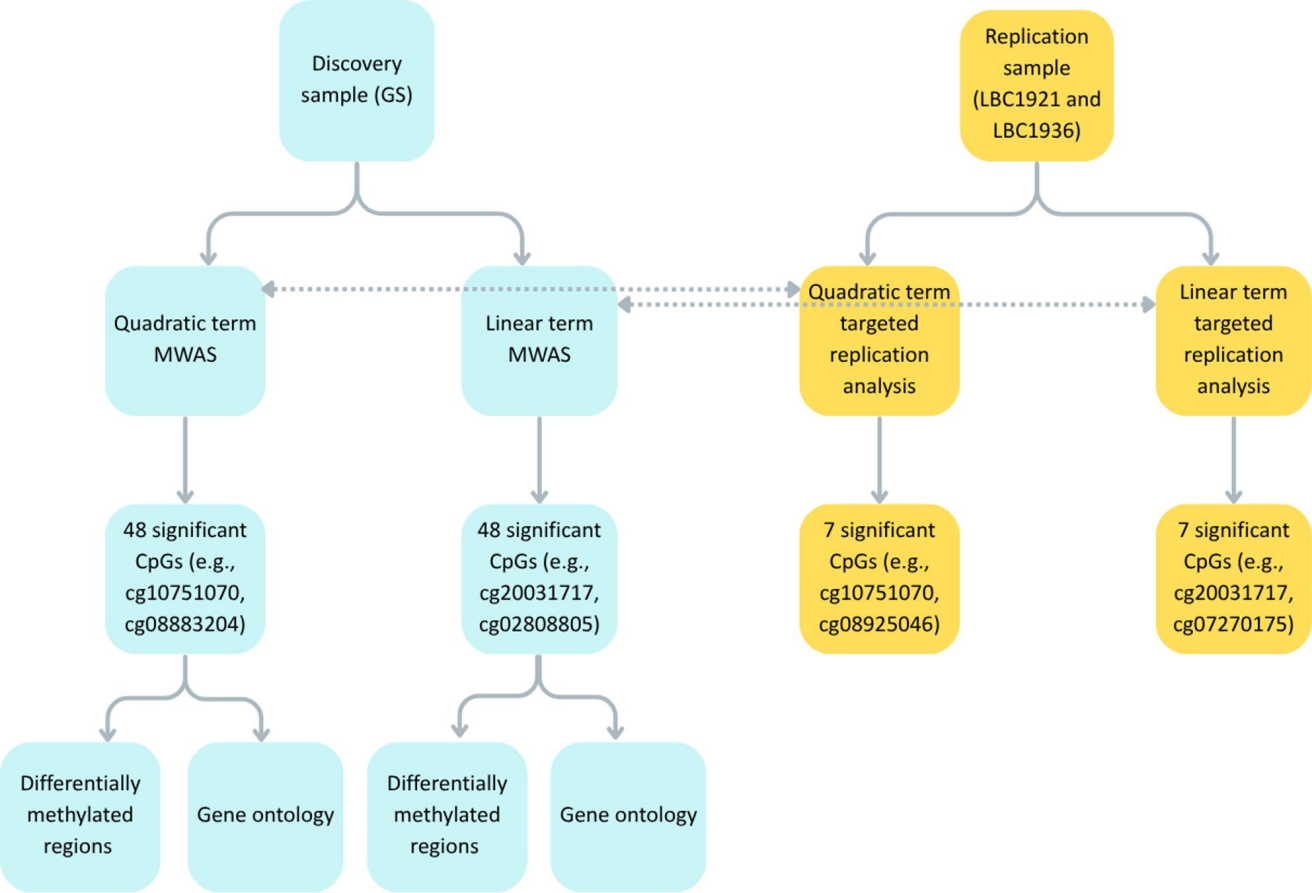
The workflow diagram of this study

The present study investigated the methylome-wide association for CYP2C19-determined metaboliser status from a large cohort, Generation Scotland (GS). Findings were replicated in Lothian Birth Cohorts 1921 and 1936. We investigated both non-linear and linear effects of CYP2C19 metaboliser status on DNA methylation. We also investigated whether the use of CYP2C19-related drugs interacted with CYP2C19 metaboliser status to affect DNA methylation. The workflow diagram of the study is shown in Fig. 1.

## Methods

### Participants

#### Generation Scotland (GS)

GS is a family-based population study that mainly consists of people with European ancestry from across Scotland. It was established in 2006 to identify the causes of common psychiatric disorders such as depression and anxiety. Blood samples collected at baseline were processed to generate genetic and DNA methylation data. Details of this cohort have been reported elsewhere [18].

## Lothian birth cohort (LBC) 1921 and LBC1936

Participants from LBC1921 and LBC1936, born in 1921 and 1936, comprise community-dwelling adults recruited from Edinburgh and surrounding Lothian area at baseline ages 79 (*N* = 550) and 70 (*N* = 1091), respectively [19, 20].

## Genetic data

Genotyping of GS was conducted on blood samples collected at baseline using IlluminaHumanOmniExpressExome-8v1.0 BeadChip (48.8%) or Illumina HumanOmniExpressExome-8 v1.2 BeadChip (51.2%) [21]. Due to the high consistency between the two versions of arrays, quality check and imputation were performed altogether across the entire cohort. Quality check is consistent with previous publications [22]. Individuals with sex mismatch, population outlier, and a call rate < 98% were excluded. SNPs with a call rate < 98%, Hardy-Weinberg equilibrium (HWE) *P* value < 1 × 10^− 6^, INFO < 0.1, and minor allele frequency (MAF) ≤ 1% were excluded. Imputation in GS was conducted using the Sanger Imputation Server with the HRC v1.1 as the reference sample [23].

Genotyping of LBC1921 and LBC1936 was performed on blood samples collected at baseline using the Illumina610-Quadv1 chip (Illumina, Inc., San Diego, CA, USA). We excluded participants with a call rate < 95%, sex mismatch, and evidence of non-European ancestry. We removed SNPs if MAF < 5%, call rate < 98%, and HWE *P v*alue < 0.001. Imputation and quality control procedures based on the INFO score followed the same approach as used in GS.

## CYP2C19 metaboliser status

We used imputed genotype data to derive CYP2C19 metaboliser status. The PGxPOP pipeline was used to assign likely metaboliser status based on diplotypes [24]. Five levels were included: poor metaboliser, intermediate metaboliser, normal metaboliser, rapid metaboliser, and ultrarapid metaboliser. Such an approach has been used in previous studies as a proxy for enzyme function and has been widely used in pharmacogenetic research and therapeutic recommendations [8, 25–27]. For example, metaboliser status inferred from CYP2C19 alleles has been associated with both treatment efficacy and tolerability of antidepressants in an Australian adult cohort [8]. Compared with normal metabolisers, poor metabolisers showed a higher efficacy, whereas rapid metabolisers demonstrated greater tolerability.

The *CYP2C19* gene is highly polymorphic with 38 variant alleles that encode CYP2C19 enzyme with no function (e.g., CYP2C19*2), decreased function (e.g., CYP2C19*9), normal function (e.g., CYP2C19*1), or increased function (e.g., CYP2C19*17). We ran PGxPOP on phased, imputed genotypes for chromosome 10 with hg19 coordinates and extracted phenotypes for the *CYP2C19* gene. Variants used to assign haplotypes were rs4244285 (CYP2C19*2), rs4986893 (CYP2C19*3), and rs12248560 (CYP2C19*17), with CYP2C19*1 as the reference. According to the diplotype to phenotype definitions, participants with two no-function alleles were categorised as “poor metabolisers” (e.g., CYP2C19*2/*2). Participants with two decreased function alleles or one normal/decreased function allele and one no-function allele were categorised as “intermediate metabolisers” (e.g., CYP2C19*1/*2). Participants with two normal function alleles or one decreased function and one increased function alleles were categorised as “normal metabolisers” (e.g., CYP2C19*1/*1. Participants with one increased function allele were categorised as “rapid metabolisers” (e.g., CYP2C19*1/*17). Participants with two increased function alleles were categorised as “ultrarapid metabolisers” (e.g., CYP2C19*17/*17). CYP2C19 metaboliser status was coded numerically (1 = poor, 2 = intermediate, 3 = normal, 4 = rapid, 5 = ultrarapid) and analysed as a continuous variable. This approach offers maximum statistical power to detect non-linear and linear trends in DNA methylation across different metaboliser groups.

### DNA methylation data

DNA methylation data of GS were generated from blood samples collected at the baseline using the Illumina MethylationEPIC array. The protocol of preprocessing and quality check has been described in detail elsewhere [28]. In short, CpG sites were removed if they had an outlying beta value (>+/-3 SD, *P* < 0.001), detection *P* value < 0.005, and low or outlying bead counts (< 3 or > 5% of the entire sample). Participants with sex mismatch with self-reported data and an outlier detection *P* < 0.01 for more than 5% of all CpG sites were removed from the analysis. Cross-hybridising and polymorphic CpG sites mapping to common SNPs (MAF > 0.05) were removed from the analysis [29]. M-values were used for further analyses. To account for relatedness, M-values were residualised against the genomic relationship matrix created using GCTA [30]. The residualised M-values of 752,741 CpG probes were then carried into MWAS [31].

DNA methylation data of LBC1921 and LBC1936 were obtained from blood samples collected at baseline using the HumanMethylation450K array. Quality control and normalisation were conducted using the R package ‘minfi’ (version 1.38.0) [32]. Probes were excluded if they had a low call rate (< 95%), outlying M-values (>+/-3 SD, *P* < 0.001), or were identified as cross-hybridising or polymorphic. We excluded participants without sufficient cell count information. All participants with methylation data were unrelated. M-value transformation was applied in the same manner as for the GS. A total of 459,309 CpG probes were available for the replication analysis.

## MWAS statistical model

A discovery MWAS was conducted in GS. Two MWAS analyses were conducted here using the Omic-data-based complex trait analysis (OSCA) software (version 2.0) [33]. We set residualised M-values as the independent variable, and the quadratic or linear term of CYP2C19 metaboliser status as the dependent variable. The first MWAS investigated the associations between the quadratic term of CYP2C19 metaboliser status and DNA methylation, and the second MWAS investigated the associations with the linear term. Examining both quadratic and linear terms enables detection of different types of association patterns. These include: (1) U-shaped or inverted U-shaped patterns (i.e., non-linear patterns) where only the quadratic term shows significant associations. In this case, individuals at both ends of the distribution (e.g., poor and ultrarapid metabolisers) show similar deviations in DNA methylation relative to normal metabolisers; (2) J-shaped or inverted J-shaped patterns where both quadratic and linear terms show significant associations. Such patterns indicate an overall linear trend, but DNA methylation is more responsive at one end of the distribution; (3) A simple linear pattern where only the linear term shows significant associations. Covariates are age, sex, first ten genetic principal components (PCs), smoking status (current/past/non-smoker), pack-years, first 20 methylation PCs, DNA methylation batch, and DNA methylation-estimated cell proportions (CD8 + T, CD4 + T, natural killer cells, B cells and granulocytes). Proportions of white blood cell types were estimated using the Houseman algorithm using the ‘minfi’ R package based on Reinius et al.’s peripheral blood reference data. Details of the implementation of this method have been described in the cohort paper [34]. These covariates were selected a priori to adjust for confounders, batch effects, and technical variations, in line with previous MWAS conducted in GS and LBC cohorts [29]. In the first MWAS which investigated the associations between the quadratic term of CYP2C19 metaboliser status and DNA methylation, we additionally adjusted for the linear term of CYP2C19 metaboliser status to isolate the non-linear effects of the quadratic term. Bonferroni correction was applied across the entire methylome, with a *P* value < 6.64 × 10^− 8^ (0.05/752,741) set as the significance threshold. Results from the linear regression model were presented as the main findings. We also used the mixed-linear-model-based method (MOA) as a secondary method to account for collinearity between CpG sites [33]. MOA incorporates a methylation omics-relatedness matrix as a random effect to control for correlations among distal CpG sites.

Given the influence of age on DNA methylation and prevalence of medication, we investigated whether the associations between CYP2C19 metaboliser status and DNA methylation varied by age in GS. We extracted M-values of significant CpG sites in each MWAS. They were included as dependent variables in linear regression models with an interaction term between the quadratic or linear term CYP2C19 metaboliser status and age. Same covariates except age were adjusted for as in the respective MWAS.

We performed a targeted replication analysis on the total sample from LBC1921 and LBC1936, adjusting for the same covariates as in the discovery MWAS except for pack-years which was unavailable. Specifically, we first identified significant CpG sites from the two discovery MWAS and were covered in the LBC cohorts. We then examined the associations between the M-values of these CpG sites and the respective quadratic or linear term of CYP2C19 metaboliser status in the LBC cohorts. A nominal significance threshold (*P* < 0.05) was used in the targeted replication analysis.

## Pathway enrichment analysis

We used the ’gometh‘ function from the ‘missmethyl’ R package for Gene Ontology (GO) and Kyoto Encyclopedia of Genes and Genomes (KEGG) [35]. Gometh analysis accounts for the number of CpG sites per gene to balance the statistical bias introduced by differing number of probes per gene present on the array, and CpGs that are annotated to multiple genes [36]. Significant CpG sites found in the above two MWAS were used as the target list and the entire EPIC array as the background list. GO terms and KEGG pathway enrichment analyses were conducted separately. Default settings were used for the analysis.

### Differentially methylated region (DMR) analysis

We conducted the DMR analysis using the ‘dmrff’ package in R [37]. DNA methylation data from a randomly selected list of participants from GS (*N* = 1,000) were used as the reference panel. Significant DMRs for the quadratic and linear terms of CYP2C19 metaboliser status were identified based on the following criteria: (1) the distance between two nearby probes within a DMR was at most 500 base pairs; (2) false discovery rate (FDR)-adjusted *P* value < 0.05, and (3) the same direction of MWAS effect estimates for the individual probes within a DMR. We used R package “coMET” [38] to visualise DNA co-methylation pattern.

### Interaction effect of CYP2C19 metaboliser status and CYP2C19-related medication use on DNA methylation

We examined the interaction effect between CYP2C19 metaboliser status and CYP2C19-metabolised medications use on DNA methylation. This is to investigate whether the effect of CYP2C19 metaboliser status on DNA methylation is driven by medication use. A list of drugs and their relationship with CYP2C19 enzyme (inducer, inhibitor, and substrate) were extracted from Drugbank (Additional file 1, also see URL: https://go.drugbank.com/bio_entities/BE0003536). Drugs associated with the *CYP2C19* gene were selected from the pharmacogenomics database. This list was used to flag records in community-dispensed prescription data (Prescribing Information System; covering 2009 to 2021, regardless of formulation, dose or prescribing duration [39]. We identified users of CYP2C19-metabolised drugs by including any participant with a flagged record less than 6 months prior to baseline assessment (*n* = 1460) to reflect recent medication use, and the remaining participants were treated as controls (*n* = 4866).

We extracted M-values of CpG sites that showed non-linear associations with CYP2C19 metaboliser status as the dependent variables given that such a pattern may indicate pharmacological implications. We checked the significance of the interaction term of CYP2C19 metaboliser status (categorised as three groups: normal, rapid/intermediate, and ultrarapid/poor) and CYP2C19-related medication use (categorised as four groups: not current user (*n* = 4866), users of drugs that CYP2C19 act as inducer (*n* = 67), inhibitor (*n* = 230), and substrate(*n* = 1163)) in linear regression models, adjusting for age, sex, first ten genetic PCs, smoking status, pack-years, first 20 methylation PCs, DNA methylation batch, and DNA methylation-estimated cell proportions as covariates. CYP2C19 metaboliser status and CYP2C19-related medication use were included in the model as nominal variables.

### Secondary hits analysis

#### mQTL analysis

Significant CpG associations with the linear term of CYP2C19 metaboliser status may be explained solely by the *cis*-mQTL effects. To investigate whether there is any secondary signal, we conducted another MWAS, adding the M-value of the top CpG site annotated to *CYP2C19* as an additional covariate. We also examined the correlations between significant CpG sites within the linkage disequilibrium (LD) block (r^2^ > 0.8) of the top mQTL of the top CpG site. This is to account for the possibility that nearby methylation sites are picking up signals from the same LD block. LD blocks were defined using European-ancestry reference data from the 1000 Genomes Project (R package “LDlinkR” [40]).

### Tissue-specific gene-expression analysis

To investigate potential differential expression genes (DEG) between blood and liver, we investigated tissue-specific gene expression among 30 tissues (GTEx v8 30 general tissue types) using the “GENE2FUNC” under the platform “FUMAGWAS” (https://fuma.ctglab.nl/gene2func). Genes of interest were genes that significant CpG sites mapped to. We used the ‘IlluminaHumanMethylationEPICmanifest’ R package (version 3.21) [41] to map CpG sites to genes. Significantly enriched gene sets in a specific tissue were defined by a two-sided t-test of gene expression per tissue versus all remaining tissues. Genes with a Bonferroni corrected *P* value < 0.05 (*P* < 0.00167) were defined as the DEG sets.

## Results

### Discovery MWAS in GS

A total of 18,396 GS participants with quality-controlled genetic and DNA methylation data were included in the MWAS (41.2% male, mean age = 47.5 years, standard deviation (SD) of age = 14.9 years, Table 1).

**Table 1:** Demographic characteristics for Generation Scotland individuals with quality-controlled genetic and DNA methylation data (*n* = 18,396)

Demographic variables	Value
*Age in years, mean (SD)*	47.5 (14.9)
*Sex*, *n (%)*	
Female	10,823 (58.8)
Male	7573 (41.2)
*Wave*, *n (%)*	
1	5086 (27.7)
2	4447 (24.2)
3	8863 (48.2)
*Smoking status*, *n (%)*	
Current smoker	3138 (17.1)
Former smoker (quitted within last 12 months)	445 (2.4)
Former smoker (quitted more than 12 months ago)	4727 (25.7)
Never smoker	9404 (51.1)
Missing	682 (3.7)
Pack years, mean (SD)	7.31 (14.11)
*CYP2C19 metaboliser status*,*n (%)*	
Poor	399 (2.2)
Intermediate	4754 (25.8)
Normal	7395 (40.2)
Rapid	4984 (27.1)
Ultrarapid	864 (4.7)

SD, standard deviation

**Fig. 2:**
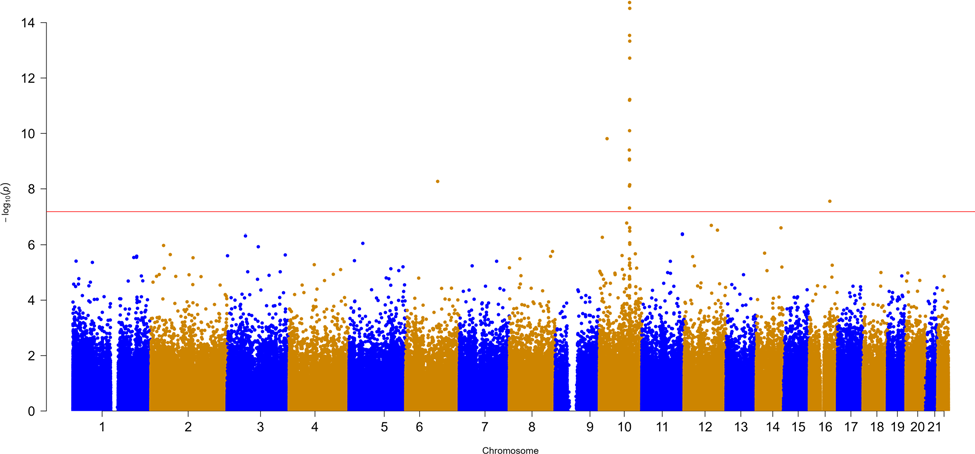
Manhattan plot for the MWAS on the quadratic term of CYP2C19 metaboliser status

**Fig. 3:**
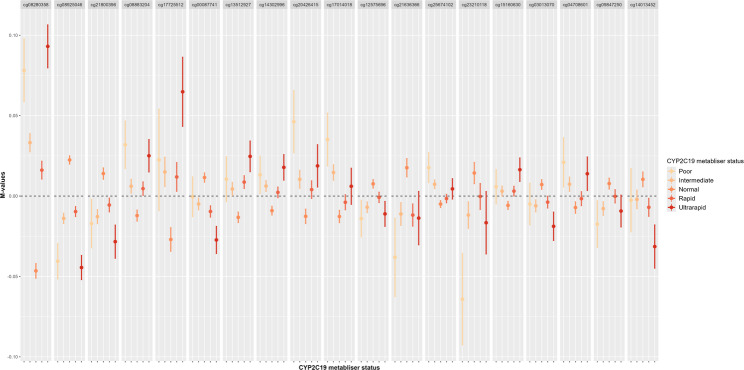
Distribution of DNA methylation for CpG sites that showed non-linear associations with CYP2C19 metaboliser status

A total of 48 CpG sites were significantly associated with the quadratic term of CYP2C19 metaboliser status with the Bonferroni threshold (*P* < 6.64 × 10^− 8^). The Manhattan plot and QQ plot are shown in Fig. 2 and Additional file 2: Figure S1, respectively. The full list of CpG sites that were significantly associated with the quadratic term of CYP2C19 metaboliser status is shown in Additional file 2: Table S1. Of these 48 CpG sites, 19 CpG sites were associated with the quadratic term of CYP2C19 metaboliser status but not with the linear term, indicating non-linear associations with CYP2C19 metaboliser status (Fig. 3; Table 2). These non-linear CpG sites are annotated to genes other than *CYP2C19* such as *TBC1D12*,* PDLIM1*, *ACSM6*, and *CYP2C18*. All these genes are located close to *CYP2C19* on chromosome 10. DNA methylation of cg08280358, cg08883204, cg17725512, cg13512927, cg14302996, cg20426415, cg17014018, cg25674102, cg15160630, and cg04708601 showed a U-shaped relationship with CYP2C19 metaboliser status where normal metaboliser status had the lowest methylation level. DNA methylation of cg08925046, cg21800396, cg00087741, cg12575696, cg21636366, cg23210118, cg03013070, cg09847250, and cg14013452 showed an inverted U-shaped relationship with CYP2C19 metaboliser status where normal metaboliser status had the highest methylation level.

**Table 2:** CpG sites with significant non-linear associations with CYP2C19 metaboliser status

CpG	CHR	BP	B	SE	*P*	Nearest gene
cg08280358	10	96189867	0.919	0.036	1.04 × 10^− 143^	*TBC1D12*
cg08925046	10	97008920	-1.420	0.065	2.97 × 10^− 106^	*PDLIM1*
cg21800396	10	96968197	-0.476	0.048	6.95 × 10^− 23^	*ACSM6*
cg08883204	10	97069028	0.474	0.050	1.14 × 10^− 21^	
cg17725512	10	96447808	0.206	0.023	6.85 × 10^− 19^	*CYP2C18*
cg00087741	10	96961488	-0.511	0.059	5.33 × 10^− 18^	*ACSM6*
cg13512927	10	95984834	0.447	0.052	1.18 × 10^− 17^	*PLCE1*
cg14302996	10	97205147	0.514	0.063	3.36 × 10^− 16^	*SORBS1*
cg20426415	10	96446783	0.301	0.038	1.88 × 10^− 15^	*CYP2C18*
cg17014018	10	96442621	0.338	0.044	2.90 × 10^− 14^	*CYP2C18*
cg12575696	10	96998139	-0.444	0.064	5.94 × 10^− 12^	*PDLIM1*
cg21636366	10	96447855	-0.195	0.030	7.97 × 10^− 11^	*CYP2C18*
cg25674102	10	24684432	0.506	0.079	1.55 × 10^− 10^	*KIAA1217*
cg23210118	10	95653378	-0.161	0.026	3.98 × 10^− 10^	*TMEM20*
cg15160630	10	96048116	0.422	0.069	8.45 × 10^− 10^	*PLCE1*
cg03013070	10	95965579	-0.347	0.057	9.04 × 10^− 10^	*PLCE1*
cg04708601	6	101880078	0.280	0.048	5.39 × 10^− 09^	*GRIK2*
cg09847250	16	65397909	-0.281	0.051	2.80 × 10^− 08^	*LOC283867*
cg14013452	10	96130481	-0.203	0.037	4.91 × 10^− 08^	

CHR, chromosome; BP, base pair; SE, standard error

**Fig. 4:**
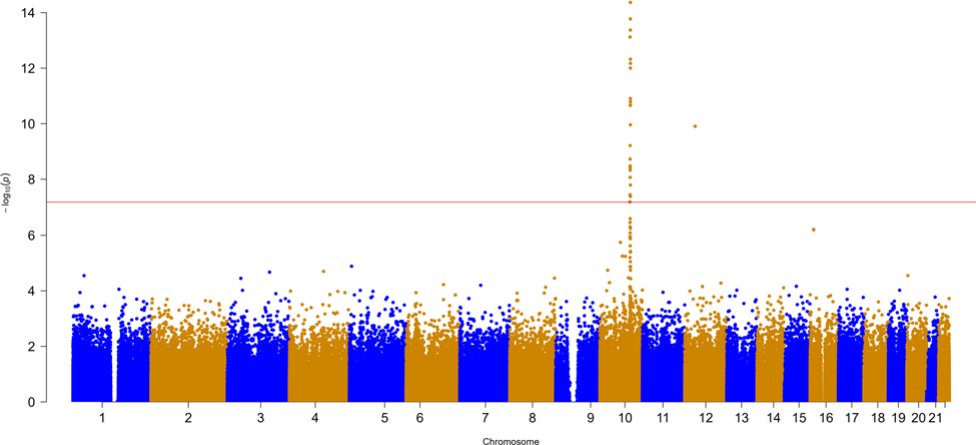
Manhattan plot for the MWAS on the linear term of CYP2C19 metaboliser status

There were also 48 CpG sites significantly associated with the linear term of CYP2C19 metaboliser status after Bonferroni correction (Additional file 2: Table S2). The Manhattan plot and QQ plot are shown in Fig. 4 and Additional file 2: Figure S2, respectively. The most significant CpG sites are annotated to *CYP2C19*,* NOC3L*, *PDLIM1*, *TBC1D12*, and *PLCE1* genes on Chromosome 10. Of these 48 CpG sites, 29 were also significant in quadratic term MWAS, with 8 CpG sites more sensitive to poor metaboliser status and 21 CpG sites more sensitive to ultrarapid metaboliser status (Additional file 2: Figures S3 and S4). 19 CpG sites were only significant in the linear term MWAS (Additional file 2: Figure S5). The MOA MWAS showed similar results to the linear model for both quadratic and linear terms of CYP2C19 metaboliser status (Additional file 2: Tables S3 and S4). The correlation of standardised regression coefficients of significant CpG sites between linear and MOA models is shown in Additional file 2: Figure S6.

We found that age only modified the association between the quadratic term of CYP2C19 metaboliser status and DNA methylation of cg10751070, with an increasing effect by age (P_FDR_ for interaction = 0.038, see Additional file 2: Figure S7). All other associations between the quadratic or linear term of CYP2C19 metaboliser status and DNA methylation were consistent across age.

### Targeted replication analysis in LBC1921 and LBC1936

**Fig. 5:**
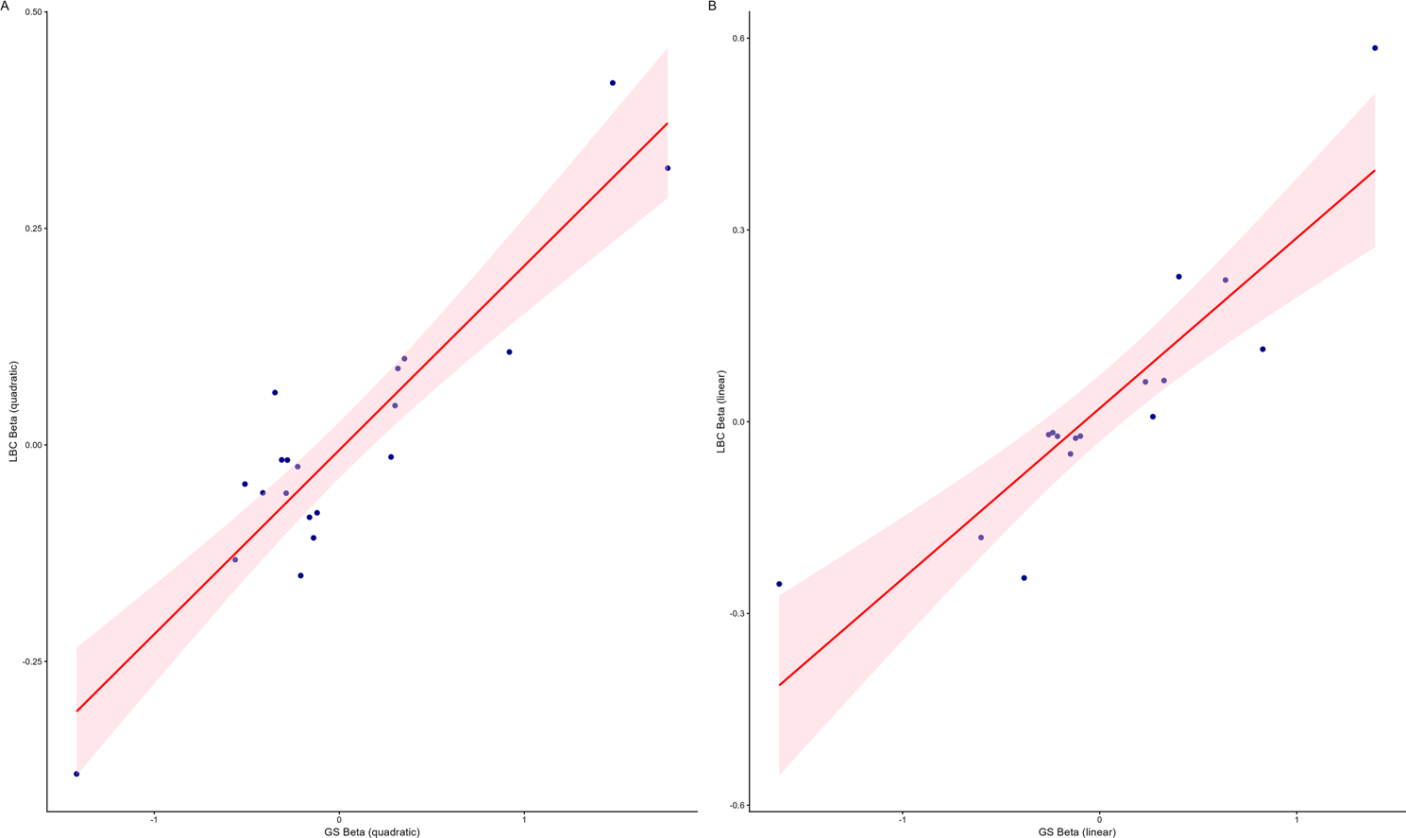
Targeted replication analysis in LBC1921 and LBC1936. **A**: Scatter plot showing the correlation of standardised regression coefficients between the discovery MWAS and the targeted replication analysis on the quadratic term of CYP2C19 metaboliser status. **B**: Scatter plot showing the correlation of standardised regression coefficients between the discovery MWAS and the targeted replication analysis on the linear term of CYP2C19 metaboliser status

A total of 1238 participants with CYP2C19 metaboliser status and DNA methylation data in the combined cohort of LBC1921 and LBC1936 were included in the targeted replication analysis (Mean age = 72.7 years (SD = 4.55), 53.2% female). Twenty CpG sites associated with the quadratic term of CYP2C19 metaboliser status and 16 CpG sites associated with the linear term in the discovery MWAS were covered in the LBC cohorts. We found 7 CpG sites associated with the quadratic term of CYP2C19 metaboliser status and 7 CpG sites associated with the linear term with nominal significance (Additional file 2: Table S5). We found that the standardised effect sizes were strongly correlated between the CpG sites that were significant in the discovery MWAS and were covered in the targeted replication analysis (quadratic term: *r* = 0.92; linear term: *r* = 0.89). Of the 20 CpG sites significantly associated with the quadratic term of CYP2C19 metaboliser status in the discovery MWAS, 18 showed consistent directions of effect in the targeted replication analysis. All 16 CpG sites that were significantly associated with the linear term in the discovery MWAS remained in the same direction of association in the targeted replication analysis (Fig. 5).

### Pathway enrichment analysis

Analysis of the GO terms showed enrichment in biological processes involving metabolic activities and the P450 pathway. There were 206 and 162 enriched GO terms for MWAS on the quadratic term and linear term of CYP2C19 metaboliser status with a *P* value < 0.05, respectively. However, none were significant after the FDR correction. The top ten GO terms are listed in Table 3. Top pathways were driven by *CYP2C19*,* CYP2C18*,* PKP2*, and *PDLIM1* genes. The test of KEGG pathways showed that pathways such as chemical carcinogenesis and metabolic pathways, including drug metabolism, were enriched. There were 9 and 11 nominally significant KEGG pathways for MWAS on the quadratic term and linear term of CYP2C19 metaboliser status with a *P* value < 0.05, respectively. There was no significant pathway after FDR correction (Table 4). Nominally significant pathways were driven by *CYP2C19*,* CYP2C18*,* PKP2*, *PDLIM1*,* ACSM6*,* and PLCE1* genes.

**Table 3:** Results for gene ontology (GO) analysis for the MWAS on the quadratic and linear terms of CYP2C19 metaboliser status at *P* threshold 6.64 × 10^− 8^. BP, biological process; MF, molecular function; DE genes, genes that are differentially methylated

CYP2C19 metaboliser status	GO ID	Ontology	Term	*N*	DE genes	*P*_DE_	*P*_FDR_
Quadratic term	GO:0008392	MF	Arachidonic acid epoxygenase activity	16	*CYP2C19*,* CYP2C18*	1.99 × 10^− 5^	0.232
	GO:0008391	MF	Arachidonic acid monooxygenase activity	18	*CYP2C19*,* CYP2C18*	2.72 × 10^− 5^	0.232
	GO:0019373	BP	Epoxygenase P450 pathway	18	*CYP2C19*,* CYP2C18*	3.09 × 10^− 5^	0.232
	GO:0070330	MF	Aromatase activity	26	*CYP2C19*,* CYP2C18*	7.47 × 10^− 5^	0.421
	GO:0019825	MF	Oxygen binding	33	*CYP2C19*,* CYP2C18*	1.23 × 10^− 4^	0.554
	GO:0016712	MF	Oxidoreductase activity, acting on paired donors, with incorporation or reduction of molecular oxygen, reduced flavin or flavoprotein as one donor, and incorporation of one atom of oxygen	43	*CYP2C19*,* CYP2C18*	1.84 × 10^− 4^	0.692
	GO:0019369	BP	Arachidonic acid metabolic process	58	*CYP2C19*,* CYP2C18*	4.4 × 10^− 4^	1
	GO:0018675	MF	(S)-limonene 6-monooxygenase activity	2	*CYP2C19*	8.23 × 10^− 4^	1
	GO:0018676	MF	(S)-limonene 7-monooxygenase activity	2	*CYP2C19*	8.23 × 10^− 4^	1
	GO:0019113	MF	Limonene monooxygenase activity	2	*CYP2C19*	8.23 × 10^− 4^	1
Linear term	GO:0008392	MF	Arachidonic acid epoxygenase activity	16	*CYP2C19*,* CYP2C18*	1.81 × 10^− 5^	0.202
	GO:0008391	MF	Arachidonic acid monooxygenase activity	18	*CYP2C19*,* CYP2C18*	2.47 × 10^− 5^	0.202
	GO:0019373	BP	Epoxygenase P450 pathway	18	*CYP2C19*,* CYP2C18*	2.68 × 10^− 5^	0.202
	GO:0070330	MF	Aromatase activity	26	*CYP2C19*,* CYP2C18*	5.90 × 10^− 5^	0.332
	GO:0019825	MF	Oxygen binding	33	*CYP2C19*,* CYP2C18*	9.80 × 10^− 5^	0.442
	GO:0016712	MF	Oxidoreductase activity, acting on paired donors, with incorporation or reduction of molecular oxygen, reduced flavin or flavoprotein as one donor, and incorporation of one atom of oxygen	43	*CYP2C19*,* CYP2C18*	1.64 × 10^− 4^	0.614
	GO:0019369	BP	Arachidonic acid metabolic process	58	*CYP2C19*,* CYP2C18*	3.52 × 10^− 4^	1
	GO:0098632	MF	Cell-cell adhesion mediator activity	52	*PKP2*,* PDLIM1*	3.74 × 10^− 4^	1
	GO:0098631	MF	Cell adhesion mediator activity	67	*PKP2*,* PDLIM1*	6.02 × 10^− 4^	1
	GO:0071614	MF	Linoleic acid epoxygenase activity	2	*CYP2C18*	7.07 × 10^− 4^	1

The top ten GO terms are listed

**Table 4:** Kyoto Encyclopedia of Genes and Genomes (KEGG) pathway analysis for the MWAS on the quadratic and linear terms of CYP2C19 metaboliser status at *P* threshold 6.64 × 10^− 8^. DE genes: genes that are differentially methylated

CYP2C19 metaboliser status	KEGG pathway ID	Description	*N*	DE genes	*P*_DE_	*P*_FDR_
Quadratic term	hsa05204	Chemical carcinogenesis - DNA adducts	70	*CYP2C19*,* CYP2C18*	0.000	0.151
	hsa04726	Serotonergic synapse	113	*CYP2C19*,* CYP2C18*	0.003	0.501
	hsa01100	Metabolic pathways	1532	*ACSM6*,* CYP2C19*,* CYP2C18*,* PLCE1*	0.010	1
	hsa00650	Butanoate metabolism	27	*ACSM6*	0.015	1
	hsa00591	Linoleic acid metabolism	30	*CYP2C19*	0.016	1
	hsa00830	Retinol metabolism	68	*CYP2C18*	0.030	1
	hsa00982	Drug metabolism - cytochrome P450	71	*CYP2C19*	0.030	1
	hsa00590	Arachidonic acid metabolism	61	*CYP2C19*	0.032	1
	hsa03320	PPAR signaling pathway	75	*SORBS1*	0.045	1
Linear term	hsa05204	Chemical carcinogenesis - DNA adducts	70	*CYP2C19*,* CYP2C18*	0.000	0.136
	hsa04726	Serotonergic synapse	113	*CYP2C19*,* CYP2C18*	0.002	0.279
	hsa01100	Metabolic pathways	1532	*ACSM6*,* CYP2C19*,* CYP2C18*,* PLCE1*	0.005	0.625
	hsa04820	Cytoskeleton in muscle cells	232	*PKP2*,* PDLIM1*	0.007	0.625
	hsa00650	Butanoate metabolism	27	*ACSM6*	0.013	0.809
	hsa00591	Linoleic acid metabolism	30	*CYP2C19*	0.013	0.809
	hsa00590	Arachidonic acid metabolism	61	*CYP2C19*	0.028	1
	hsa00830	Retinol metabolism	68	*CYP2C18*	0.029	1
	hsa00982	Drug metabolism - cytochrome P450	71	*CYP2C19*	0.029	1
	hsa00562	Inositol phosphate metabolism	73	*PLCE1*	0.041	1
	hsa05412	Arrhythmogenic right ventricular cardiomyopathy	86	*PKP2*	0.048	1

KEGG pathways with P_DE_ < 0.05 are listed

### DMR analysis

Table 5 shows that 3 DMRs annotated to *NOC3L* (10:96123159–96123172), *PLCE1* (10:96048116–96048482), and *SORBS1* (10:97204891–97205147) were associated with the quadratic term of CYP2C19 metaboliser status at FDR-adjusted *P* value < 0.05. A total of 5 DMRs annotated to *PDLIM1* (10:97051104–97051225; 97051255–97051319), *NOC3L* (10:96121776–96121853), *CYP2C18* (10:96442621–96443071), and an open-sea area (10:96928199–96928657) were identified as associated with the linear term of CYP2C19 metaboliser status at FDR-adjusted *P* value < 0.05. No significant DMR was identified in the *CYP2C19* gene. We plotted the methylation pattern of all CpG sites annotated to *NOC3L* given the limited number of CpG sites within this locus allows for clear visualisation of co-methylation pattern (Additional file 2: Figure S8).

**Table 5:** Significant differentially methylated regions identified from the MWAS on the quadratic and linear terms of CYP2C19 metaboliser status

CYP2C19 metaboliser status	CHR	Start	End	Gene	Effect	SE	Adjusted *P* values	CpGs
Quadratic term	10	96123159	96123172	*NOC3L*	− 1.357	0.061	1.50 × 10^− 104^	cg07889765, cg08923894
	10	96048116	96048482	*PLCE1*	2.592	0.369	1.62 × 10^− 06^	cg15160630, cg10680511
	10	97204891	97205147	*SORBS1*	1.726	0.204	2.08 × 10^− 11^	cg03799335, cg14302996
Linear term	10	97051104	97051225	*PDLIM1*	− 1.234	0.141	1.39 × 10^− 12^	cg11911874, cg03178678
	10	97051255	97051319	*PDLIM1*	− 0.888	0.133	1.89 × 10^− 05^	cg06542614, cg05599883
	10	96121776	96121853	*NOC3L*	0.261	0.036	6.16 × 10^− 07^	cg15209556, cg17162216, cg10164249
	10	96442621	96443071	*CYP2C18*	− 0.660	0.116	0.01	cg17014018, cg04501839, cg19852607
	10	96928199	96928657		1.224	0.182	1.28 × 10^− 05^	cg25102879, cg24087710

SE: standard error

We investigated the interaction effect of CYP2C19 metaboliser status and CYP2C19-metabolised medication use on M-values of CpG sites that showed significant non-linear associations with CYP2C19 metaboliser status. No significant interaction effect was detected (*P* values for interaction term after FDR correction: all > 0.05, Additional file 2: Table S6), indicating that the association between CYP2C19 metaboliser status and DNA methylation did not vary by CYP2C19-metabolised medication use.

### Secondary hits analysis

When additionally adjusting for the M-value of the CpG that was most robustly associated with the linear term of CYP2C19 metaboliser status (cg20031717), the associations between other significant CpG probes (e.g., cg02808805, cg00051662) annotated to the *CYP2C19* gene and CYP2C19 metaboliser status were attenuated but still significant (Additional file 2: Table S7), indicating that associations between CYP2C19 metaboliser status and DNA methylation were not explained solely from mQTL of the *CYP2C19* gene. Six CpG sites (cg24087710, cg01529847, cg02989450, cg14196507, cg27423310, cg10304160) were no longer significant, whilst 2 CpG sites (cg00087741, cg05771722) became significant. Forty-one CpG sites remained significant. We found four other CpG sites (cg00051662, cg02808805, cg14196507, cg15851404) within the LD block of the top mQTL (rs200889969) of cg20031717. Correlations between these CpG sites were low (coefficients ranged between − 0.18 and 0.24, Additional file 2: Figure S9).

We tested the DEG of annotated genes of significant non-linear CpG sites (*TBC1D12*, *PDLIM1*, *ACSM6*, *CYP2C18*, *PLCE1*, *SORBS1*, *KIAA1217*, *TMEM20*,* GRIK2*, *and LOC283867*). None of these genes expressed differently between blood and liver tissues (all *P*-values > 0.00167 i.e., − log10 *P* value < 2.78, Additional file 2: Figure S10).

## Discussion

We found that CYP2C19 metaboliser status had both non-linear and linear effects on widespread DNA methylation in a large population health dataset. DNA methylation associations were consistent in an independent dataset. Significant CpGs were enriched in metabolic pathways involving P450 epoxygenase and drug metabolism, driven by multiple genes. No interactive effect was observed between CYP2C19-related medication use and CYP2C19 metaboliser status on DNA methylation.

Few studies have directly examined the non-linear relationship between CYP2C19 metaboliser status and blood biomarkers, yet evidence suggests differences between normal metabolism and abnormal metabolism (ultra-rapid or poor). For example, research on specific CYP2C19 genotypes has shown that CYP2C19*17 homozygous carriers demonstrate enhanced/rapid CYP2C19 activity, potentially leading to reduced antidepressant response [9]. Similarly, poor and intermediate metabolisers experience increased adverse effects from antidepressant use compared to normal metabolisers [25]. Additionally, a non-linear relationship between CYP2C19-inferred metabolising speed was associated with plasma C-reactive protein (CRP) levels, a protein closely linked to inflammatory mechanisms underlying depression [42].

Among the CpG sites showing non-linear relationships with CYP2C19 metaboliser status, hypermethylation of cg21800396 and cg00087741 and hypomethylation of cg08883204, cg14302996, cg25674102, and cg04708601 were observed in normal metaboliser status and are also associated with a higher level of CRP and increased risk of Type 2 diabetes in other MWAS [43, 44]. Normal metaboliser status was also associated with hypermethylation of cg09847250 and hypomethylation of cg17014018 which are related to a lower level of CRP. However, neither of these two CpG sites is linked to Type 2 diabetes. These findings indicate that CYP2C19 metaboliser status may have implications for inflammation, and normal metaboliser status may link to increased risk of Type 2 diabetes. Notably, 2 non-linear CpG signals are located on Chromosome 6 (cg04708601) and 16 (cg09847250), indicating that CYP2C19 metaboliser status affects distal DNA methylation. Annotated genes (*GRIK2*,* LOC283867*) are associated with response to antidepressant treatment [45, 46]. Both cg04708601 and cg09847250 were replicated in the LBC cohorts.

Significant CpG sites and DMRs are mapped to multiple genes on Chromosome 10, such as *TBC1D12*,* NOC3L*, *PLCE1*,* SORBS1*,* PDLIM1*,* and CYP2C18*. Of these, *SORBS1* is specifically relevant to non-linear CpG signal (cg14302996) and DMR. The protein encoded by this gene has functions in insulin signalling and stimulation, where dysfunction may be associated with insulin resistance and Type 2 diabetes [47]. The top non-linear CpG signal (cg08280358, replicated in the LBC cohorts) is annotated to *TBC1D12*, which is associated with metabolites of warfarin (an anticoagulant) and clopidogrel (an antiplatelet) [48, 49]. *NOC3L and PLCE1* are associated with low-density lipoprotein (LDL) cholesterol levels in the blood [50]. Previous research highlights the important role CYP enzymes play in the metabolism of cholesterols [51]. This explains that lipid profile, including LDL subfractions, predicts response to antidepressants, as found by another study [52]. *PDLIM1* has been associated with metabolite levels of warfarin and steroid hormone levels (biosynthesis promoted by CYP) [48, 53]. *CYP2C18* is another CYP superfamily member associated with metabolite levels of warfarin and clopidogrel as well as serum metabolites [48, 49, 54].

It is possible that some CYP2C19-related medications, especially antidepressants, are more prevalent in older people. However, effect modification of age on the associations between CYP2C19 metaboliser status and DNA methylation was only evident for one CpG (cg10751070, replicated in the LBC cohorts). This CpG did not demonstrate a non-linear association with CYP2C19 metaboliser status, suggesting that age-related medication use is unlikely to drive our findings.

We did not detect the interaction effect between CYP2C19-related medication use and CYP2C19 metaboliser status on DNA methylation. This indicates that metabolites of these drugs and metabolic status determined by *CYP2C19* variants may affect DNA methylation independently. This is partly supported by previous MWAS in European ancestries on antidepressant use [55, 56]. CpG signals associated with antidepressant use found in these two studies do not overlap with any significant CpG sites or DMRs in our study, indicating that CYP2C19 metaboliser status and antidepressant use may not share biological pathways. However, this has not formally been tested with other CYP2C19-metabolised drugs, e.g., warfarin, clopidogrel, to our knowledge.

Strengths of our study include that GS is the largest single sample MWAS to date, which allows adequate power to detect individual CpG sites and DMRs associated with CYP2C19 metaboliser status. Additionally, as the metaboliser status of CYP2C19 enzyme is genetically determined, it is not subject to reverse causation or confounding. This approach is suitable for large population-based studies and clinical practice, particularly where direct measurement of enzymatic activity is not feasible. However, our study has some limitations. First, our analyses only included participants predominantly with European ancestry, so our findings may not be generalisable to individuals of other ancestries. Second, DNA methylation from the whole blood may not be representative of DNA methylation from the liver where the *CYP2C19* gene is mostly expressed and may obscure findings from individual cell types. There is a lack of evidence on the concordance of DNA methylation profile in relation to CYP enzymes between blood and liver tissues. However, our tissue-specific gene expression analysis suggests that genes to which significant non-linear CpG sites mapped do not exhibit differential gene expression between blood and liver tissues. Direct comparison of epigenetic variation between liver and whole blood is difficult due to a lack of large-scale multi-tissue samples. Cross-tissue DNA methylation may be an important area for future development. Third, information on drug use is only available in a subset of participants who gave consent for data linkage to electronic health records, which might limit the power to detect a significant interaction effect. Future studies should gather well-powered health record data with more detailed information on medication use (e.g., dose, side effects, switching) to assess how CYP2C19 metaboliser status influences medication use. This will inform the clinical utility of DNA methylation for guiding the design of therapeutic strategies beyond the genetically determined enzymatic activity. Finally, CYP2C19 enzymatic activity may be influenced by wider environmental factors such as drug use, inflammation, and diet, which are not captured by genetic variants. DNA methylation is also influenced by wider social and environmental factors such as psychosocial stress, socioeconomic status, and lifestyle factors. Future studies should integrate these measures to disentangle the roles of social, genetic, and environmental factors on DNA methylation.

In conclusion, this MWAS in a large-scale cohort suggests that genetically-determined CYP2C19 metaboliser status is associated with both local and distal DNA methylation. Our mQTL analysis and the non-linear CpG signals found in MWAS indicate that the effects of CYP2C19 metaboliser status on DNA methylation are not driven by mQTL. These signals have biological implications relevant to the response of certain drugs, inflammation, lipid levels, and Type 2 diabetes. The use of CYP2C19-metabolised medications did not interact with CYP2C19 metaboliser status to affect DNA methylation, indicating that CYP2C19 metaboliser status may impact DNA methylation and metabolic pathways that do not depend on the use of medications metabolised by this enzyme. Future studies should confirm our findings in other populations and investigate how CYP2C19 metaboliser status interacts with detailed medication records to influence DNA methylation and health consequences.

## Supplementary Information

Below is the link to the electronic supplementary material.

Additional file 1

Additional file 2

## Data Availability

According to the terms of consent for GS participants, access to individual-level genetic, DNA methylation data and phenotypes need to be approved by the GS Access Committee (https://www.ed.ac.uk/generation-scotland/for-researchers/access). Application should be made to access@generationscotland.org. Data dictionary for GS is available at https://datashare.ed.ac.uk/handle/10283/2988. LBC1921 and LBC1936 data access must be approved by the LBC research team. LBC data access guideline is in the study website at https://lothian-birth-cohorts.ed.ac.uk/data-access-collaboration.

## Abbreviations

CYP2C19: The CYP family 2 subfamily C, polypeptide 19
CpG: Cytosine-phosphate-guanine
CRP: C-reactive protein
DEG: Differential expression genes
DMR: Differentially methylated region
FDR: False discovery rate
GO: Gene ontology
GS: Generation Scotland
HWE: Hardy-Weinberg equilibrium
KEGG: Kyoto encyclopedia of genes and genomes
LBC: Lothian birth cohort
LD: Linkage disequilibrium
MAF: Minor allele frequency
MOA: Mixed-linear-model-based omic association
mQTL: Methylation quantitative trait loci
MWAS: Methylome-wide association study
OSCA: Omic-data-based complex trait analysis
PC: Principal component
SD: Standard deviation
