# Supplementary material for: The non-linear and linear effects of CYP2C19 metaboliser status on DNA methylation: a methylome-wide association study
Source: Clin Epigenetics. 2026 Apr 22;18:111. doi: 10.1186/s13148-026-02125-w (PMC13251042; doi:10.1186/s13148-026-02125-w)
Supplement: Supplementary file 2 — Additional file 2 [file 13148_2026_2125_MOESM1_ESM.docx]

Figure S1 QQ plot for the MWAS on the quadratic term of CYP2C19 metaboliser status.





Figure S2 QQ plot for the MWAS on the linear term of CYP2C19 metaboliser status.





Figure S3 Distribution of DNA methylation for CpG sites that were more sensitive to poor CYP2C19 metaboliser status.


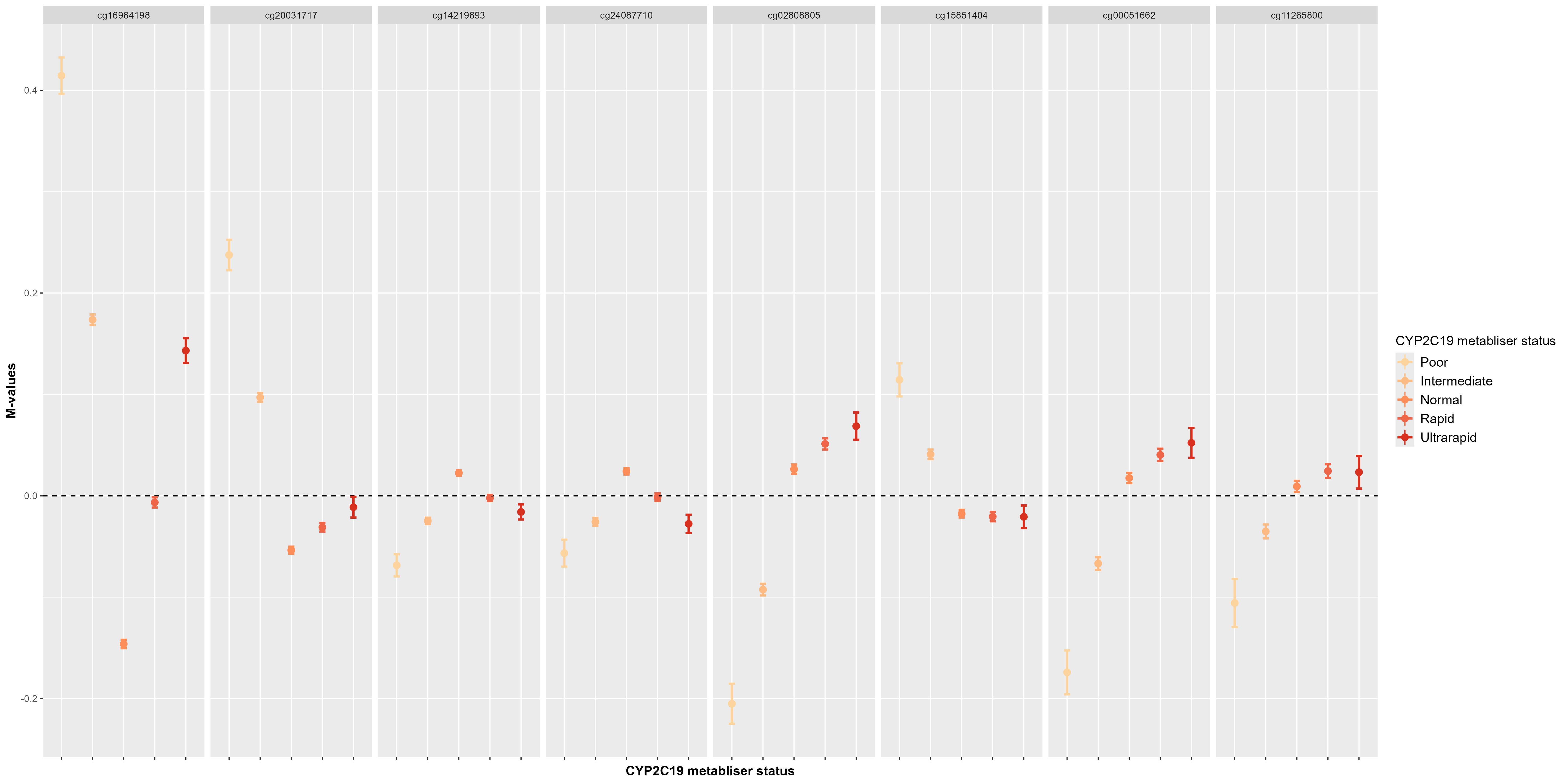


Figure S4 Distribution of DNA methylation for CpG sites that were more sensitive to ultrarapid CYP2C19 metaboliser status.


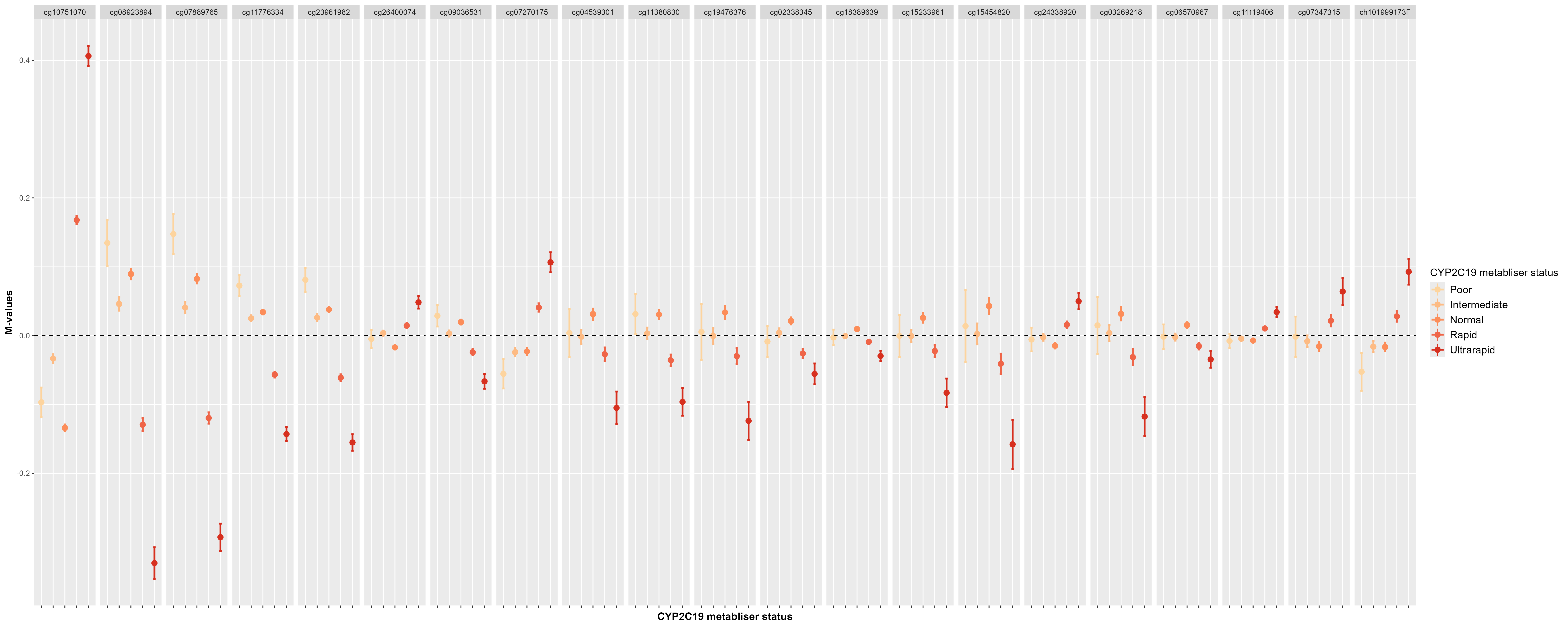


Figure S5 Distribution of DNA methylation for CpG sites that showed linear associations with CYP2C19 metaboliser status.


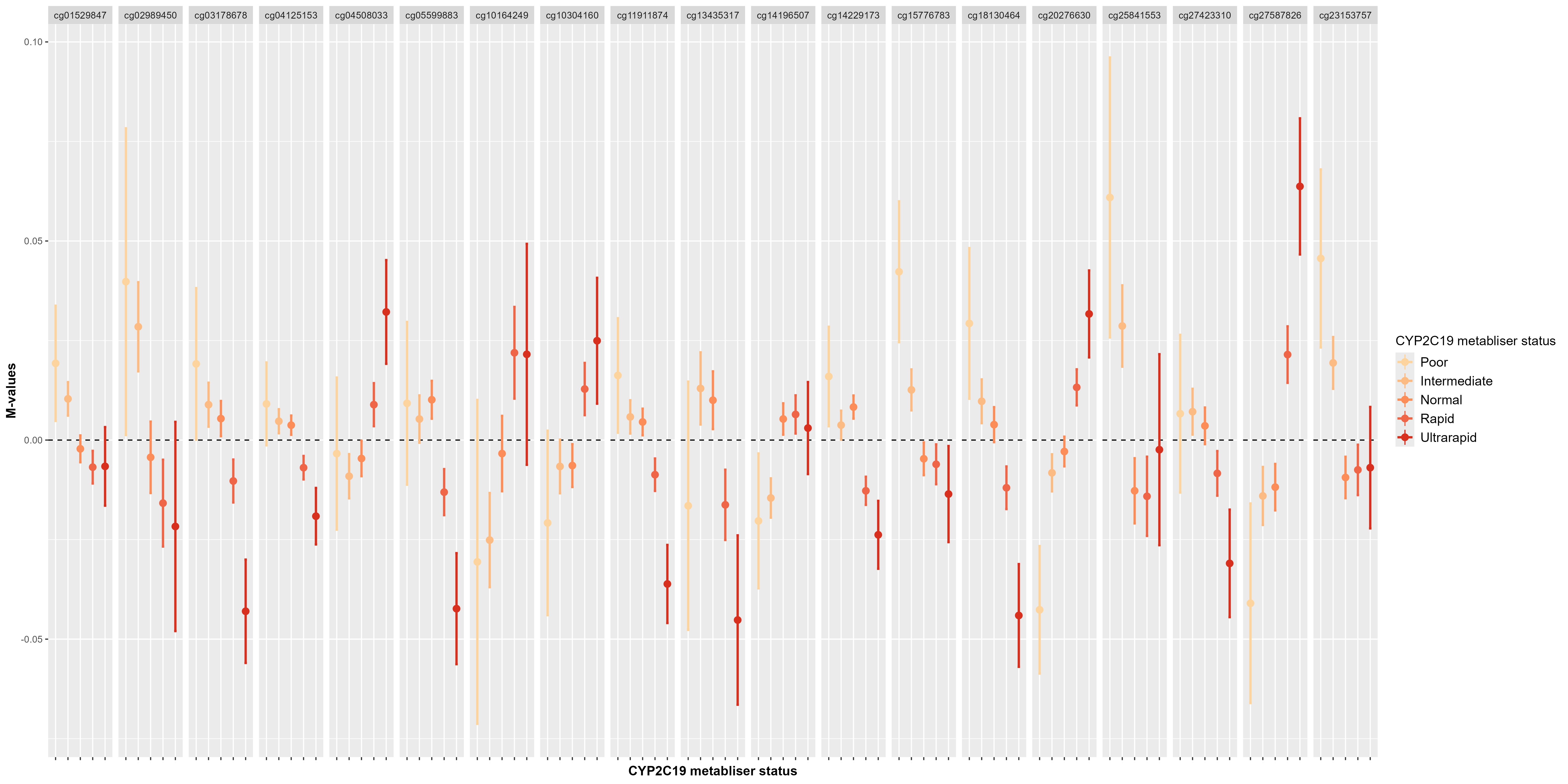


Figure S6 Scatter plots for the correlation of standardised regression coefficients of significant CpG sites between linear and MOA model. A: MWAS on the quadratic term of CYP2C19 metaboliser status. B: MWAS on the linear term of CYP2C19 metaboliser status


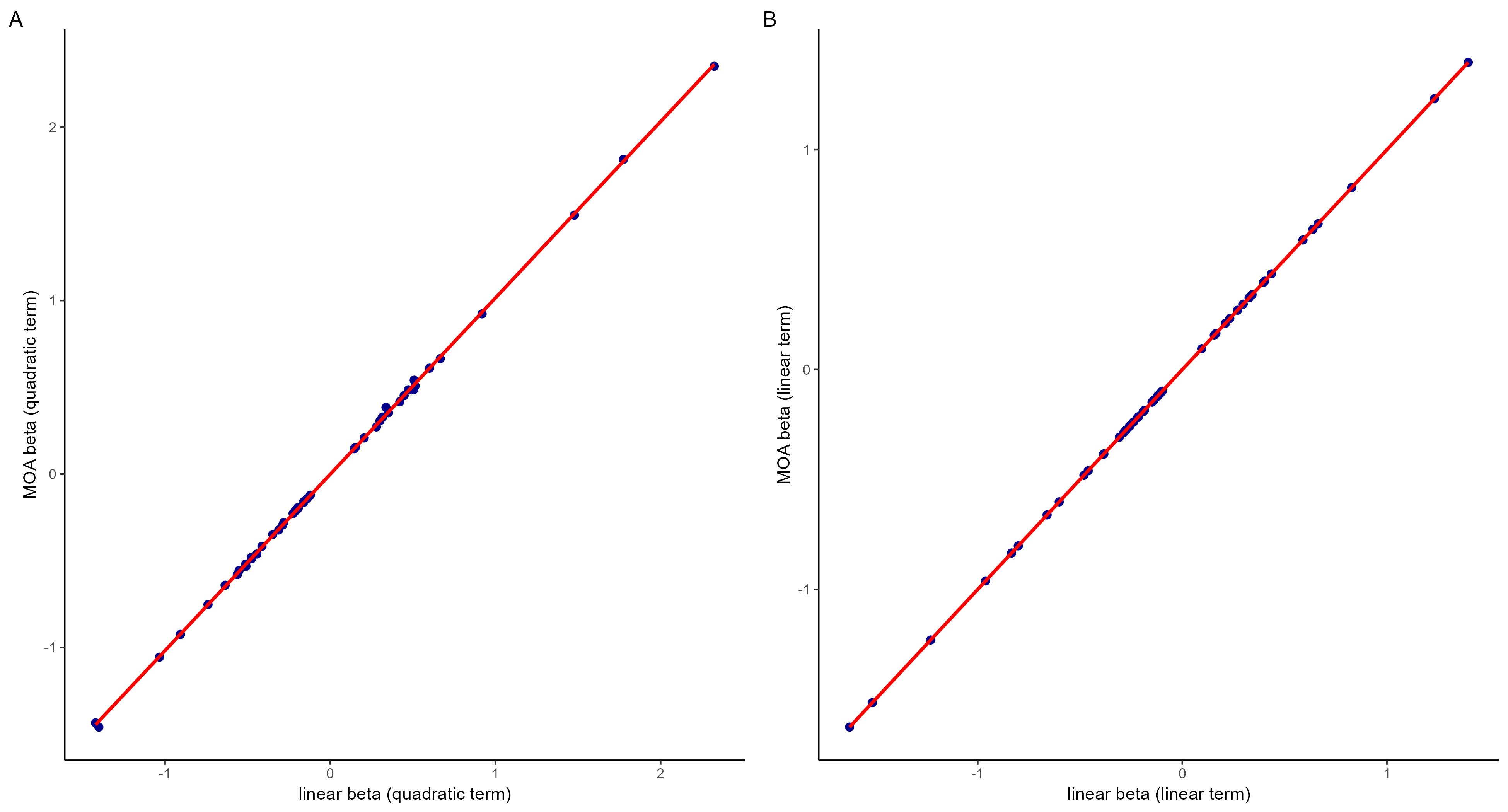


Figure S7 Association between the quadratic term of CYP2C19 metaboliser status and DNA methylation of cg10751070 by age


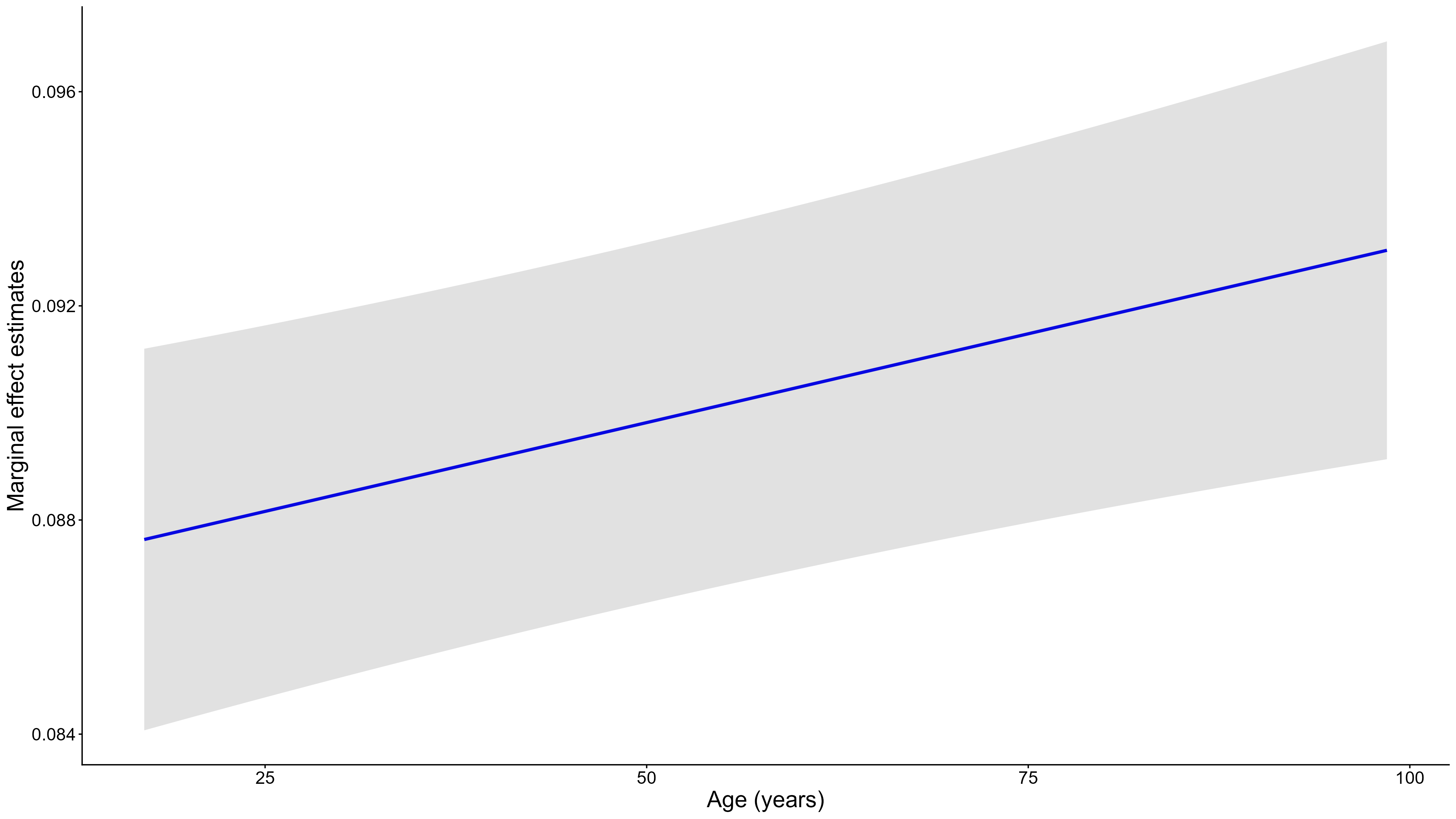


Figure S8 DNA co-methylation pattern of CpG sites annotated to *NOC3L*


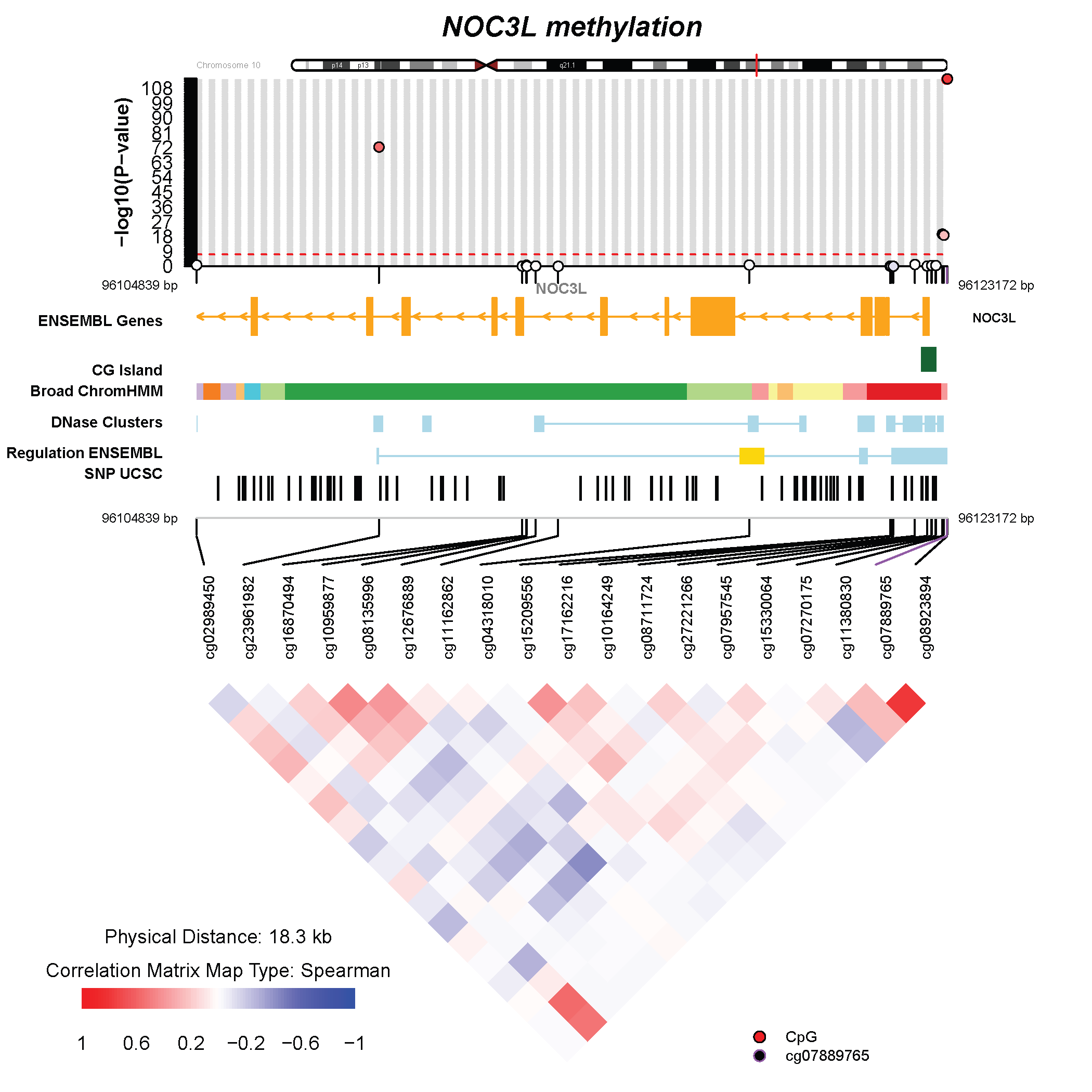


Figure S9 Correlation heatmap for CpG sites within the LD block of the nearest mQTL (rs200889969) to cg20031717


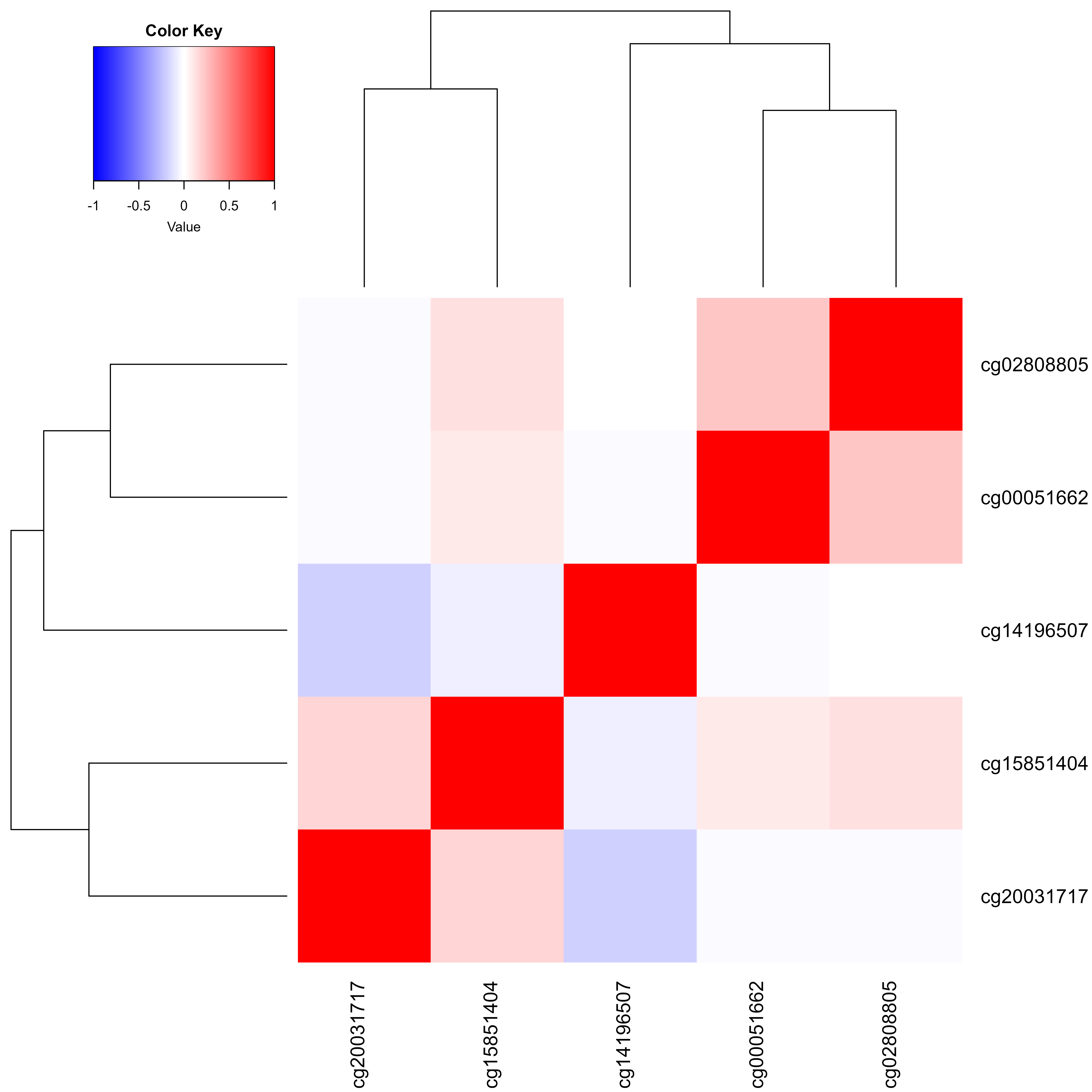


Figure S10 Results of tissue-specificity gene expression test based on the list of annotated genes of significant non-linear CpG sites.


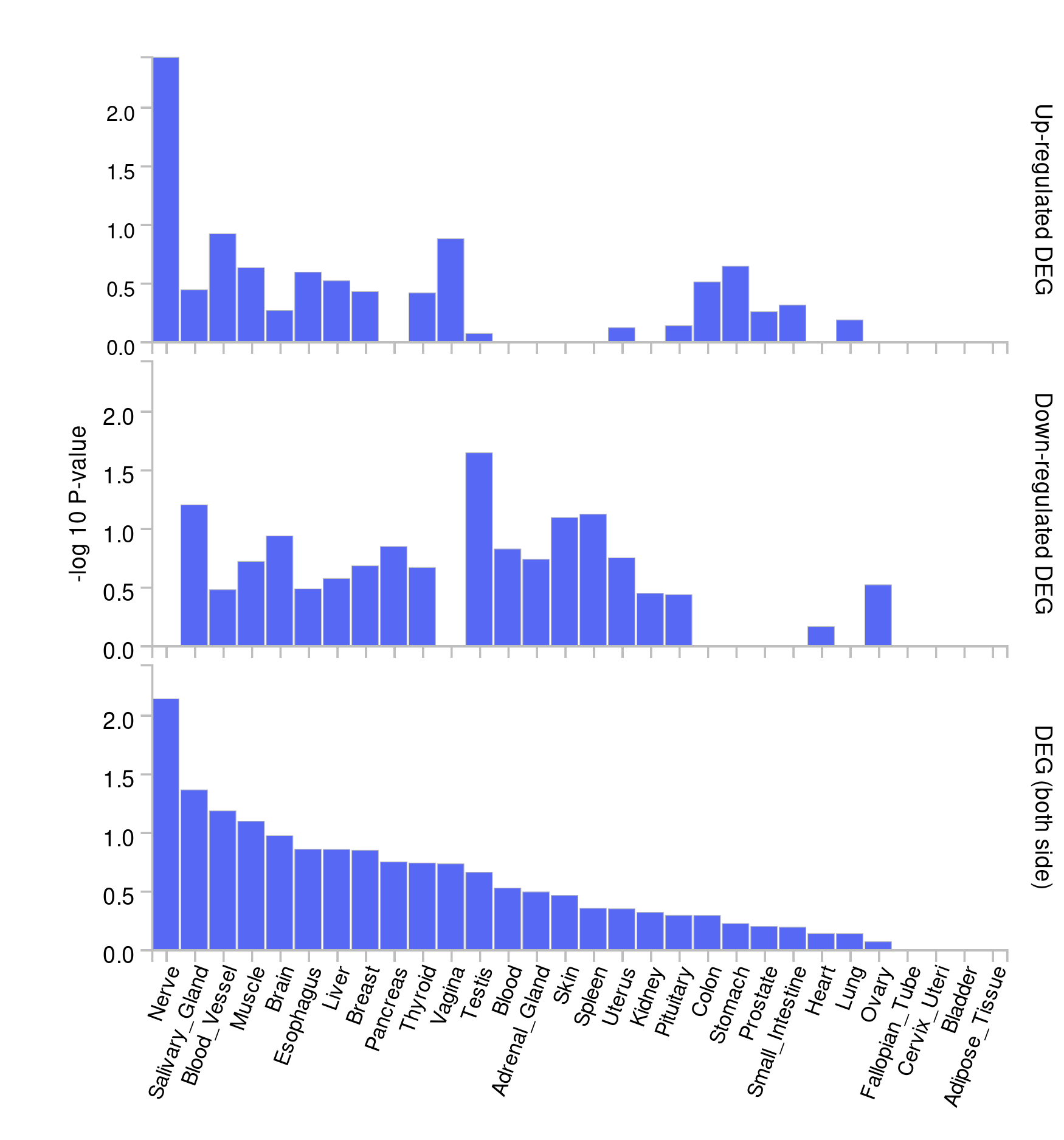


DEG: differential expression genes

Table S1 Significant CpG sites associated with the quadratic term of CYP2C19 metaboliser status.

| CpG | CHR | BP | B | SE | P | Nearest gene |
| --- | --- | --- | --- | --- | --- | --- |
| cg10751070 | 10 | 96143568 | 1.478 | 0.028 | <2.22x10^-308^ |  |
| cg16964198 | 10 | 96199371 | 2.325 | 0.028 | <2.22x10^-308^ | *TBC1D12* |
| cg20031717 | 10 | 96523248 | 1.775 | 0.044 | <2.22x10^-308^ | *CYP2C19* |
| cg08280358 | 10 | 96189867 | 0.919 | 0.036 | 1.04x10^-143^ | *TBC1D12* |
| cg08923894 | 10 | 96123172 | -0.478 | 0.021 | 1.47x10^-114^ | *NOC3L* |
| cg07889765 | 10 | 96123159 | -0.551 | 0.024 | 2.53x10^-114^ | *NOC3L* |
| cg08925046 | 10 | 97008920 | -1.420 | 0.065 | 2.97x10^-106^ | *PDLIM1* |
| cg14219693 | 10 | 96928076 | -1.400 | 0.065 | 1.56x10^-102^ |  |
| cg11776334 | 10 | 96046836 | -0.906 | 0.047 | 6.35x10^-82^ | *PLCE1* |
| cg24087710 | 10 | 96928657 | -1.033 | 0.054 | 6.54x10^-81^ |  |
| cg23961982 | 10 | 96109291 | -0.740 | 0.041 | 3.47x10^-73^ | *NOC3L* |
| cg02808805 | 10 | 96521820 | -0.636 | 0.036 | 3.93x10^-68^ | *CYP2C19* |
| cg15851404 | 10 | 96643549 | 0.602 | 0.044 | 8.19x10^-42^ |  |
| cg00051662 | 10 | 96521086 | -0.413 | 0.034 | 1.06x10^-34^ | *CYP2C19* |
| cg26400074 | 10 | 96996533 | 0.666 | 0.055 | 6.29x10^-34^ |  |
| cg09036531 | 10 | 96991505 | -0.510 | 0.047 | 1.24x10^-27^ |  |
| cg21800396 | 10 | 96968197 | -0.476 | 0.048 | 6.95x10^-23^ | *ACSM6* |
| cg08883204 | 10 | 97069028 | 0.474 | 0.050 | 1.14x10^-21^ |  |
| cg07270175 | 10 | 96123049 | 0.317 | 0.034 | 2.36x10^-20^ | *NOC3L* |
| cg04539301 | 10 | 96990923 | -0.193 | 0.021 | 2.49x10^-20^ |  |
| cg11380830 | 10 | 96123085 | -0.225 | 0.025 | 1.08x10^-19^ | *NOC3L* |
| cg19476376 | 10 | 96990921 | -0.161 | 0.018 | 3.43x10^-19^ |  |
| cg17725512 | 10 | 96447808 | 0.206 | 0.023 | 6.85x10^-19^ | *CYP2C18* |
| cg02338345 | 10 | 97036527 | -0.287 | 0.033 | 1.69x10^-18^ | *PDLIM1* |
| cg18389639 | 10 | 97049610 | -0.562 | 0.065 | 3.16x10^-18^ | *PDLIM1* |
| cg15233961 | 10 | 96990543 | -0.208 | 0.024 | 4.87x10^-18^ |  |
| cg00087741 | 10 | 96961488 | -0.511 | 0.059 | 5.33x10^-18^ | *ACSM6* |
| cg15454820 | 10 | 96990858 | -0.120 | 0.014 | 6.99x10^-18^ |  |
| cg13512927 | 10 | 95984834 | 0.447 | 0.052 | 1.18x10^-17^ | *PLCE1* |
| cg24338920 | 10 | 96075700 | 0.352 | 0.042 | 7.15x10^-17^ | *PLCE1* |
| cg14302996 | 10 | 97205147 | 0.514 | 0.063 | 3.36x10^-16^ | *SORBS1* |
| cg20426415 | 10 | 96446783 | 0.301 | 0.038 | 1.88x10^-15^ | *CYP2C18* |
| cg03269218 | 10 | 96990700 | -0.139 | 0.018 | 3.07x10^-15^ |  |
| cg17014018 | 10 | 96442621 | 0.338 | 0.044 | 2.90x10^-14^ | *CYP2C18* |
| cg06570967 | 10 | 96989650 | -0.311 | 0.041 | 4.68x10^-14^ |  |
| cg11119406 | 10 | 96881724 | 0.508 | 0.069 | 1.92x10^-13^ |  |
| cg12575696 | 10 | 96998139 | -0.444 | 0.064 | 5.94x10^-12^ | *PDLIM1* |
| cg11265800 | 10 | 96312726 | -0.212 | 0.031 | 6.34x10^-12^ | *HELLS* |
| cg21636366 | 10 | 96447855 | -0.195 | 0.030 | 7.97x10^-11^ | *CYP2C18* |
| cg25674102 | 10 | 24684432 | 0.506 | 0.079 | 1.55x10^-10^ | *KIAA1217* |
| cg23210118 | 10 | 95653378 | -0.161 | 0.026 | 3.98x10^-10^ | *TMEM20* |
| cg15160630 | 10 | 96048116 | 0.422 | 0.069 | 8.45x10^-10^ | *PLCE1* |
| cg03013070 | 10 | 95965579 | -0.347 | 0.057 | 9.04x10^-10^ | *PLCE1* |
| cg04708601 | 6 | 101880078 | 0.280 | 0.048 | 5.39x10^-09^ | *GRIK2* |
| cg07347315 | 10 | 97003301 | 0.145 | 0.025 | 7.12x10^-09^ | *PDLIM1* |
| ch.10.1999173F | 10 | 95990357 | 0.153 | 0.027 | 7.97x10^-09^ | *PLCE1* |
| cg09847250 | 16 | 65397909 | -0.281 | 0.051 | 2.80x10^-08^ | *LOC283867* |
| cg14013452 | 10 | 96130481 | -0.203 | 0.037 | 4.91x10^-08^ |  |

CHR: chromosome; BP: base pair; SE: standard error

Table S2 Significant CpG sites associated with the linear term of CYP2C19 metaboliser status.

| CpG | CHR | BP | B | SE | P | Nearest gene |
| --- | --- | --- | --- | --- | --- | --- |
| **cg20031717** | 10 | 96523248 | -1.626 | 0.039 | <2.22x10^-308^ | *CYP2C19* |
| **cg02808805** | 10 | 96521820 | 1.231 | 0.031 | <2.22x10^-308^ | *CYP2C19* |
| **cg07889765** | 10 | 96123159 | -0.802 | 0.021 | <2.22x10^-308^ | *NOC3L* |
| **cg10751070** | 10 | 96143568 | 1.397 | 0.023 | <2.22x10^-308^ |  |
| **cg11776334** | 10 | 96046836 | -1.515 | 0.041 | 7.06x10^-303^ | *PLCE1* |
| **cg08923894** | 10 | 96123172 | -0.661 | 0.018 | 1.63x10^-287^ | *NOC3L* |
| **cg23961982** | 10 | 96109291 | -1.230 | 0.036 | 1.10x10^-262^ | *NOC3L* |
| **cg16964198** | 10 | 96199371 | -0.961 | 0.028 | 2.83x10^-253^ | *TBC1D12* |
| **cg00051662** | 10 | 96521086 | 0.827 | 0.030 | 1.50x10^-170^ | *CYP2C19* |
| **cg15851404** | 10 | 96643549 | -0.834 | 0.040 | 2.67x10^-96^ |  |
| **cg07270175** | 10 | 96123049 | 0.638 | 0.031 | 3.85x10^-95^ | *NOC3L* |
| **cg11265800** | 10 | 96312726 | 0.397 | 0.028 | 3.47x10^-45^ | *HELLS* |
| **cg09036531** | 10 | 96991505 | -0.602 | 0.043 | 4.23x10^-45^ |  |
| **ch.10.1999173F** | 10 | 95990357 | 0.297 | 0.024 | 1.55x10^-34^ | *PLCE1* |
| **cg11380830** | 10 | 96123085 | -0.238 | 0.023 | 8.54x10^-26^ | *NOC3L* |
| **cg11119406** | 10 | 96881724 | 0.663 | 0.063 | 1.00x10^-25^ |  |
| cg27587826 | 10 | 96166801 | 0.270 | 0.027 | 2.84x10^-24^ | *TBC1D12* |
| **cg14219693** | 10 | 96928076 | 0.589 | 0.060 | 1.58x10^-22^ |  |
| cg20276630 | 10 | 97055439 | 0.402 | 0.042 | 7.25x10^-22^ |  |
| cg18130464 | 10 | 96039752 | -0.308 | 0.035 | 2.48x10^-18^ | *PLCE1* |
| cg14229173 | 10 | 95974688 | -0.460 | 0.053 | 5.73x10^-18^ | *PLCE1* |
| **cg26400074** | 10 | 96996533 | 0.435 | 0.050 | 6.20x10^-18^ |  |
| **cg02338345** | 10 | 97036527 | -0.259 | 0.030 | 6.80x10^-18^ | *PDLIM1* |
| **cg24338920** | 10 | 96075700 | 0.326 | 0.039 | 3.83x10^-17^ | *PLCE1* |
| cg11911874 | 10 | 97051104 | -0.386 | 0.046 | 9.44x10^-17^ | *PDLIM1* |
| cg03178678 | 10 | 97051225 | -0.275 | 0.035 | 4.28x10^-15^ | *PDLIM1* |
| **cg15454820** | 10 | 96990858 | -0.098 | 0.013 | 1.68x10^-14^ |  |
| **cg03269218** | 10 | 96990700 | -0.122 | 0.016 | 4.24x10^-14^ |  |
| cg04125153 | 10 | 95987486 | -0.481 | 0.064 | 7.53x10^-14^ | *PLCE1* |
| **cg19476376** | 10 | 96990921 | -0.120 | 0.017 | 4.75x10^-13^ |  |
| **cg04539301** | 10 | 96990923 | -0.138 | 0.019 | 6.71x10^-13^ |  |
| **cg07347315** | 10 | 97003301 | 0.164 | 0.023 | 9.88x10^-13^ | *PDLIM1* |
| **cg24087710** | 10 | 96928657 | 0.341 | 0.050 | 1.24x10^-11^ |  |
| cg15776783 | 10 | 96974685 | -0.255 | 0.038 | 1.61x10^-11^ | *ACSM6* |
| cg05599883 | 10 | 97051319 | -0.219 | 0.033 | 2.13x10^-11^ | *PDLIM1* |
| **cg15233961** | 10 | 96990543 | -0.148 | 0.022 | 2.18x10^-11^ |  |
| **cg18389639** | 10 | 97049610 | -0.383 | 0.059 | 1.09x10^-10^ | *PDLIM1* |
| cg23153757 | 12 | 33048710 | -0.192 | 0.030 | 1.24x10^-10^ | *PKP2* |
| cg01529847 | 10 | 96161365 | -0.286 | 0.046 | 6.09x10^-10^ | *TBC1D12* |
| cg04508033 | 10 | 96047603 | 0.210 | 0.035 | 1.87x10^-09^ | *PLCE1* |
| cg02989450 | 10 | 96104839 | -0.103 | 0.017 | 3.32x10^-09^ | *NOC3L* |
| cg14196507 | 10 | 96443383 | 0.232 | 0.039 | 3.83x10^-09^ | *CYP2C18* |
| cg25841553 | 10 | 96356520 | -0.111 | 0.019 | 4.70x10^-09^ | *HELLS* |
| cg10164249 | 10 | 96121853 | 0.095 | 0.016 | 8.55x10^-09^ | *NOC3L* |
| **cg06570967** | 10 | 96989650 | -0.214 | 0.038 | 1.61x10^-08^ |  |
| cg13435317 | 10 | 95826508 | -0.118 | 0.021 | 3.62x10^-08^ | *PLCE1* |
| cg27423310 | 10 | 97051091 | -0.185 | 0.034 | 4.13x10^-08^ | *PDLIM1* |
| cg10304160 | 10 | 95457300 | 0.156 | 0.029 | 6.58x10^-08^ | *FRA10AC1* |

CHR: chromosome; BP: base pair; SE: standard error. CpG sites in bold are also significant in quadratic term MWAS.

Table S3 Significant CpG sites associated with the quadratic term of CYP2C19 metaboliser status using the mixed-linear-model-based method.

| CpG | CHR | BP | B | SE | P | Nearest gene |
| --- | --- | --- | --- | --- | --- | --- |
| cg10751070 | 10 | 96143568 | 1.492 | 0.031 | <2.22x10^-308^ |  |
| cg16964198 | 10 | 96199371 | 2.350 | 0.033 | <2.22x10^-308^ | *TBC1D12* |
| cg20031717 | 10 | 96523248 | 1.814 | 0.047 | <2.22x10^-308^ | *CYP2C19* |
| cg08280358 | 10 | 96189867 | 0.923 | 0.037 | 9.37X10^-138^ | *TBC1D12* |
| cg08923894 | 10 | 96123172 | -0.482 | 0.021 | 1.20X10^-112^ | *NOC3L* |
| cg07889765 | 10 | 96123159 | -0.557 | 0.025 | 1.38X10^-112^ | *NOC3L* |
| cg14219693 | 10 | 96928076 | -1.459 | 0.067 | 9.50X10^-105^ |  |
| cg08925046 | 10 | 97008920 | -1.435 | 0.066 | 2.24X10^-104^ | *PDLIM1* |
| cg11776334 | 10 | 96046836 | -0.925 | 0.048 | 2.55X10^-82^ | *PLCE1* |
| cg24087710 | 10 | 96928657 | -1.056 | 0.055 | 2.90X10^-81^ |  |
| cg23961982 | 10 | 96109291 | -0.753 | 0.041 | 1.05X10^-73^ | *NOC3L* |
| cg02808805 | 10 | 96521820 | -0.641 | 0.037 | 3.20X10^-67^ | *CYP2C19* |
| cg15851404 | 10 | 96643549 | 0.610 | 0.045 | 1.59X10^-41^ |  |
| cg00051662 | 10 | 96521086 | -0.417 | 0.034 | 1.06X10^-34^ | *CYP2C19* |
| cg26400074 | 10 | 96996533 | 0.665 | 0.055 | 4.36X10^-33^ |  |
| cg09036531 | 10 | 96991505 | -0.533 | 0.048 | 5.91X10^-29^ |  |
| cg21800396 | 10 | 96968197 | -0.489 | 0.049 | 3.42X10^-23^ | *ACSM6* |
| cg08883204 | 10 | 97069028 | 0.485 | 0.050 | 4.20X10^-22^ |  |
| cg07270175 | 10 | 96123049 | 0.328 | 0.035 | 2.27X10^-21^ | *NOC3L* |
| cg04539301 | 10 | 96990923 | -0.197 | 0.021 | 1.80X10^-20^ |  |
| cg11380830 | 10 | 96123085 | -0.228 | 0.025 | 7.58X10^-20^ | *NOC3L* |
| cg19476376 | 10 | 96990921 | -0.164 | 0.018 | 2.74X10^-19^ |  |
| cg17725512 | 10 | 96447808 | 0.208 | 0.023 | 6.91X10^-19^ | *CYP2C18* |
| cg02338345 | 10 | 97036527 | -0.294 | 0.033 | 8.46X10^-19^ | *PDLIM1* |
| cg18389639 | 10 | 97049610 | -0.580 | 0.066 | 8.79X10^-19^ | *PDLIM1* |
| cg15233961 | 10 | 96990543 | -0.212 | 0.024 | 4.36X10^-18^ |  |
| cg15454820 | 10 | 96990858 | -0.122 | 0.014 | 5.30X10^-18^ |  |
| cg00087741 | 10 | 96961488 | -0.519 | 0.060 | 7.81X10^-18^ | *ACSM6* |
| cg13512927 | 10 | 95984834 | 0.452 | 0.054 | 3.85X10^-17^ | *PLCE1* |
| cg24338920 | 10 | 96075700 | 0.353 | 0.042 | 1.04X10^-16^ | *PLCE1* |
| cg17014018 | 10 | 96442621 | 0.384 | 0.047 | 3.06X10^-16^ | *CYP2C18* |
| cg20426415 | 10 | 96446783 | 0.307 | 0.038 | 1.37X10^-15^ | *CYP2C18* |
| cg03269218 | 10 | 96990700 | -0.142 | 0.018 | 2.20X10^-15^ |  |
| cg14302996 | 10 | 97205147 | 0.507 | 0.064 | 2.44X10^-15^ | *SORBS1* |
| cg06570967 | 10 | 96989650 | -0.323 | 0.042 | 8.86X10^-15^ |  |
| cg11119406 | 10 | 96881724 | 0.541 | 0.071 | 2.63X10^-14^ |  |
| cg12575696 | 10 | 96998139 | -0.462 | 0.065 | 1.61X10^-12^ | *PDLIM1* |
| cg11265800 | 10 | 96312726 | -0.213 | 0.031 | 7.79X10^-12^ | *HELLS* |
| cg21636366 | 10 | 96447855 | -0.194 | 0.030 | 1.29X10^-10^ | *CYP2C18* |
| cg23210118 | 10 | 95653378 | -0.160 | 0.026 | 5.88X10^-10^ | *TMEM20* |
| cg15160630 | 10 | 96048116 | 0.417 | 0.070 | 2.35X10^-09^ | *PLCE1* |
| cg03013070 | 10 | 95965579 | -0.348 | 0.059 | 3.05X10^-09^ | *PLCE1* |
| cg25674102 | 10 | 24684432 | 0.486 | 0.082 | 3.54X10^-09^ | *KIAA1217* |
| ch.10.1999173F | 10 | 95990357 | 0.154 | 0.027 | 8.79X10^-09^ | *PLCE1* |
| cg07347315 | 10 | 97003301 | 0.146 | 0.025 | 9.11X10^-09^ | *PDLIM1* |
| cg04708601 | 6 | 101880078 | 0.271 | 0.049 | 3.04X10^-08^ | *GRIK2* |
| cg14013452 | 10 | 96130481 | -0.206 | 0.038 | 4.09X10^-08^ |  |
| cg09847250 | 16 | 65397909 | -0.279 | 0.051 | 4.43X10^-08^ | *LOC283867* |
| cg04164578 | 10 | 96798765 | 0.292 | 0.054 | 6.44X10^-08^ | *CYP2C8* |

CHR: chromosome; BP: base pair; SE: standard error

Table S4 Significant CpG sites associated with the linear term of CYP2C19 metaboliser status using the mixed-linear-model-based method.

| CpG | CHR | BP | B | SE | P | Nearest gene |
| --- | --- | --- | --- | --- | --- | --- |
| cg20031717 | 10 | 96523248 | -1.626 | 0.040 | <2.22x10^-308^ | *CYP2C19* |
| cg02808805 | 10 | 96521820 | 1.231 | 0.032 | <2.22x10^-308^ | *CYP2C19* |
| cg07889765 | 10 | 96123159 | -0.802 | 0.021 | <2.22x10^-308^ | *NOC3L* |
| cg10751070 | 10 | 96143568 | 1.397 | 0.025 | <2.22x10^-308^ |  |
| cg11776334 | 10 | 96046836 | -1.515 | 0.042 | 1.76x10^-291^ | *PLCE1* |
| cg08923894 | 10 | 96123172 | -0.661 | 0.019 | 1.01x10^-277^ | *NOC3L* |
| cg23961982 | 10 | 96109291 | -1.230 | 0.036 | 2.84x10^-255^ | *NOC3L* |
| cg16964198 | 10 | 96199371 | -0.961 | 0.029 | 1.09x10^-246^ | *TBC1D12* |
| cg00051662 | 10 | 96521086 | 0.827 | 0.030 | 1.59x10^-169^ | *CYP2C19* |
| cg15851404 | 10 | 96643549 | -0.834 | 0.040 | 1.70x10^-97^ |  |
| cg07270175 | 10 | 96123049 | 0.638 | 0.031 | 2.38x10^-96^ | *NOC3L* |
| cg11265800 | 10 | 96312726 | 0.397 | 0.028 | 2.58x10^-46^ | *HELLS* |
| cg09036531 | 10 | 96991505 | -0.602 | 0.042 | 3.15x10^-46^ |  |
| ch.10.1999173F | 10 | 95990357 | 0.297 | 0.024 | 1.75x10^-35^ | *PLCE1* |
| cg11380830 | 10 | 96123085 | -0.238 | 0.022 | 1.51x10^-26^ | *NOC3L* |
| cg11119406 | 10 | 96881724 | 0.663 | 0.062 | 1.78x10^-26^ |  |
| cg27587826 | 10 | 96166801 | 0.270 | 0.026 | 5.49x10^-25^ | *TBC1D12* |
| cg14219693 | 10 | 96928076 | 0.589 | 0.059 | 3.39x10^-23^ |  |
| cg20276630 | 10 | 97055439 | 0.402 | 0.041 | 1.61x10^-22^ |  |
| cg18130464 | 10 | 96039752 | -0.308 | 0.035 | 6.91x10^-19^ | *PLCE1* |
| cg14229173 | 10 | 95974688 | -0.460 | 0.052 | 1.63x10^-18^ | *PLCE1* |
| cg26400074 | 10 | 96996533 | 0.435 | 0.050 | 1.77x10^-18^ |  |
| cg02338345 | 10 | 97036527 | -0.259 | 0.030 | 1.95x10^-18^ | *PDLIM1* |
| cg24338920 | 10 | 96075700 | 0.326 | 0.038 | 1.15x10^-17^ | *PLCE1* |
| cg11911874 | 10 | 97051104 | -0.386 | 0.046 | 2.92x10^-17^ | *PDLIM1* |
| cg03178678 | 10 | 97051225 | -0.275 | 0.034 | 1.48x10^-15^ | *PDLIM1* |
| cg15454820 | 10 | 96990858 | -0.098 | 0.013 | 6.05x10^-15^ |  |
| cg03269218 | 10 | 96990700 | -0.122 | 0.016 | 1.57x10^-14^ |  |
| cg04125153 | 10 | 95987486 | -0.481 | 0.063 | 2.84x10^-14^ | *PLCE1* |
| cg19476376 | 10 | 96990921 | -0.120 | 0.016 | 1.90x10^-13^ |  |
| cg04539301 | 10 | 96990923 | -0.138 | 0.019 | 2.71x10^-13^ |  |
| cg07347315 | 10 | 97003301 | 0.164 | 0.023 | 4.03x10^-13^ | *PDLIM1* |
| cg24087710 | 10 | 96928657 | 0.341 | 0.049 | 5.48x10^-12^ |  |
| cg15776783 | 10 | 96974685 | -0.255 | 0.037 | 7.16x10^-12^ | *ACSM6* |
| cg05599883 | 10 | 97051319 | -0.219 | 0.032 | 9.55x10^-12^ | *PDLIM1* |
| cg15233961 | 10 | 96990543 | -0.148 | 0.022 | 9.79x10^-12^ |  |
| cg18389639 | 10 | 97049610 | -0.383 | 0.058 | 5.16x10^-11^ | *PDLIM1* |
| cg23153757 | 12 | 33048710 | -0.192 | 0.029 | 5.88x10^-11^ | *PKP2* |
| cg01529847 | 10 | 96161365 | -0.286 | 0.045 | 3.05x10^-10^ | *TBC1D12* |
| cg04508033 | 10 | 96047603 | 0.210 | 0.034 | 9.72x10^-10^ | *PLCE1* |
| cg02989450 | 10 | 96104839 | -0.103 | 0.017 | 1.75x10^-09^ | *NOC3L* |
| cg14196507 | 10 | 96443383 | 0.232 | 0.039 | 2.03x10^-09^ | *CYP2C18* |
| cg25841553 | 10 | 96356520 | -0.111 | 0.019 | 2.51x10^-09^ | *HELLS* |
| cg10164249 | 10 | 96121853 | 0.095 | 0.016 | 4.66x10^-09^ | *NOC3L* |
| cg06570967 | 10 | 96989650 | -0.214 | 0.037 | 8.97x10^-09^ |  |
| cg13435317 | 10 | 95826508 | -0.118 | 0.021 | 2.07x10^-08^ | *PLCE1* |
| cg27423310 | 10 | 97051091 | -0.185 | 0.033 | 2.37x10^-08^ | *PDLIM1* |
| cg10304160 | 10 | 95457300 | 0.156 | 0.028 | 3.83x10^-08^ | *FRA10AC1* |

CHR: chromosome; BP: base pair; SE: standard error

Table S5 Targeted replication analysis in the LBC cohorts

| Term | CpG | CHR | BP | B | SE | P |
| --- | --- | --- | --- | --- | --- | --- |
| Quadratic term | cg10751070 | 10 | 96143568 | 0.418 | 0.044 | 8.99x10^-22^ |
|  | cg20031717 | 10 | 96523248 | 0.320 | 0.063 | 4.64x10^-07^ |
|  | cg08925046 | 10 | 97008920 | -0.380 | 0.078 | 1.05x10^-06^ |
|  | cg15454820 | 10 | 96990858 | -0.078 | 0.020 | 9.52x10^-05^ |
|  | cg03269218 | 10 | 96990700 | -0.107 | 0.028 | 1.43x10^-04^ |
|  | cg15233961 | 10 | 96990543 | -0.151 | 0.044 | 0.001 |
|  | cg08280358 | 10 | 96189867 | 0.107 | 0.045 | 0.017 |
|  | cg24338920 | 10 | 96075700 | 0.100 | 0.053 | 0.058 |
|  | cg07270175 | 10 | 96123049 | 0.088 | 0.051 | 0.082 |
|  | cg23210118 | 10 | 95653378 | -0.084 | 0.051 | 0.099 |
|  | cg02338345 | 10 | 97036527 | -0.056 | 0.041 | 0.170 |
|  | cg18389639 | 10 | 97049610 | -0.133 | 0.099 | 0.179 |
|  | cg11380830 | 10 | 96123085 | -0.025 | 0.019 | 0.184 |
|  | cg00051662 | 10 | 96521086 | -0.055 | 0.043 | 0.200 |
|  | cg20426415 | 10 | 96446783 | 0.045 | 0.056 | 0.420 |
|  | cg09036531 | 10 | 96991505 | -0.045 | 0.057 | 0.432 |
|  | cg03013070 | 10 | 95965579 | 0.060 | 0.084 | 0.474 |
|  | cg09847250 | 16 | 65397909 | -0.017 | 0.033 | 0.596 |
|  | cg06570967 | 10 | 96989650 | -0.017 | 0.036 | 0.633 |
|  | cg04708601 | 6 | 101880078 | -0.014 | 0.043 | 0.747 |
| Linear term | cg10751070 | 10 | 96143568 | 0.585 | 0.032 | 3.44x10^-76^ |
|  | cg07270175 | 10 | 96123049 | 0.222 | 0.045 | 7.29x10^-07^ |
|  | cg20031717 | 10 | 96523248 | -0.254 | 0.057 | 7.46x10^-06^ |
|  | cg20276630 | 10 | 97055439 | 0.227 | 0.055 | 3.59x10^-05^ |
|  | cg09036531 | 10 | 96991505 | -0.181 | 0.051 | 3.97x10^-04^ |
|  | cg00051662 | 10 | 96521086 | 0.113 | 0.039 | 0.003 |
|  | cg18389639 | 10 | 97049610 | -0.244 | 0.088 | 0.006 |
|  | cg24338920 | 10 | 96075700 | 0.064 | 0.047 | 0.173 |
|  | cg15233961 | 10 | 96990543 | -0.050 | 0.039 | 0.202 |
|  | cg15454820 | 10 | 96990858 | -0.022 | 0.018 | 0.215 |
|  | cg14196507 | 10 | 96443383 | 0.062 | 0.051 | 0.224 |
|  | cg11380830 | 10 | 96123085 | -0.017 | 0.017 | 0.314 |
|  | cg03269218 | 10 | 96990700 | -0.026 | 0.026 | 0.314 |
|  | cg06570967 | 10 | 96989650 | -0.023 | 0.032 | 0.484 |
|  | cg27587826 | 10 | 96166801 | 0.008 | 0.014 | 0.565 |
|  | cg02338345 | 10 | 97036527 | -0.020 | 0.037 | 0.580 |

CHR: chromosome; BP: base pair; SE: standard error

This targeted replication analysis focused on 20 CpG sites associated with the quadratic term of CYP2C19 metaboliser status and 16 CpG sites associated with the linear term in the discovery MWAS.

Table S6 Significance of the interaction effects of the CYP2C19 metaboliser status and CYP2C19-metabolised medication use on M-values of significant non-linear CpG sites.

| CpG | P_interaction_ | P_FDR_ |
| --- | --- | --- |
| cg08280358 | 0.405 | 0.640 |
| cg08925046 | 0.021 | 0.147 |
| cg21800396 | 0.059 | 0.226 |
| cg08883204 | 0.399 | 0.640 |
| cg17725512 | 0.025 | 0.147 |
| cg00087741 | 0.526 | 0.714 |
| cg13512927 | 0.671 | 0.750 |
| cg14302996 | 0.031 | 0.147 |
| cg20426415 | 0.665 | 0.750 |
| cg17014018 | 0.455 | 0.665 |
| cg12575696 | 0.169 | 0.458 |
| cg21636366 | 0.214 | 0.509 |
| cg25674102 | 0.331 | 0.628 |
| cg23210118 | 0.773 | 0.773 |
| cg15160630 | 0.285 | 0.601 |
| cg03013070 | 0.118 | 0.375 |
| cg04708601 | 0.014 | 0.147 |
| cg09847250 | 0.743 | 0.773 |
| cg14013452 | 0.588 | 0.745 |

P_interaction_: P values for the interaction term of CYP2C19 metaboliser status (categorised as three groups: normal, rapid/intermediate, and ultrarapid/poor) and CYP2C19-related medication use (categorised as four groups: not current user, inducer, inhibitor, and substrate); P_FDR_: P values for interaction term after FDR correction

Table S7 Significant CpG sites associated with the linear term of CYP2C19 metaboliser status after additional adjustment for the M-value of cg20031717.

| CpG | CHR | BP | B | SE | P | Nearest gene |
| --- | --- | --- | --- | --- | --- | --- |
| cg07889765 | 10 | 96123159 | -0.776 | 0.020 | <2.22x10^-308^ | *NOC3L* |
| cg10751070 | 10 | 96143568 | 1.404 | 0.022 | <2.22x10^-308^ |  |
| cg11776334 | 10 | 96046836 | -1.461 | 0.039 | 5.15x10^-310^ | *PLCE1* |
| cg08923894 | 10 | 96123172 | -0.643 | 0.017 | 5.89x10^-300^ | *NOC3L* |
| cg23961982 | 10 | 96109291 | -1.210 | 0.034 | 7.24x10^-281^ | *NOC3L* |
| cg02808805 | 10 | 96521820 | 1.059 | 0.030 | 5.35x10^-269^ | *CYP2C19* |
| cg00051662 | 10 | 96521086 | 0.695 | 0.029 | 7.95x10^-130^ | *CYP2C19* |
| cg16964198 | 10 | 96199371 | -0.659 | 0.029 | 3.48x10^-115^ | *TBC1D12* |
| cg07270175 | 10 | 96123049 | 0.636 | 0.029 | 3.95x10^-104^ | *NOC3L* |
| cg15851404 | 10 | 96643549 | -0.767 | 0.038 | 1.79x10^-89^ |  |
| cg09036531 | 10 | 96991505 | -0.636 | 0.041 | 3.81x10^-55^ |  |
| cg11265800 | 10 | 96312726 | 0.330 | 0.027 | 1.89x10^-34^ | *HELLS* |
| ch.10.1999173F | 10 | 95990357 | 0.275 | 0.023 | 1.46x10^-32^ | *PLCE1* |
| cg26400074 | 10 | 96996533 | 0.514 | 0.048 | 1.14x10^-26^ |  |
| cg11119406 | 10 | 96881724 | 0.636 | 0.060 | 5.27x10^-26^ |  |
| cg27587826 | 10 | 96166801 | 0.260 | 0.025 | 9.80x10^-25^ | *TBC1D12* |
| cg11380830 | 10 | 96123085 | -0.207 | 0.022 | 1.06x10^-21^ | *NOC3L* |
| cg14229173 | 10 | 95974688 | -0.482 | 0.051 | 2.34x10^-21^ | *PLCE1* |
| cg24338920 | 10 | 96075700 | 0.346 | 0.037 | 7.37x10^-21^ | *PLCE1* |
| cg15454820 | 10 | 96990858 | -0.113 | 0.012 | 1.49x10^-20^ |  |
| cg03269218 | 10 | 96990700 | -0.140 | 0.015 | 8.39x10^-20^ |  |
| cg02338345 | 10 | 97036527 | -0.256 | 0.029 | 3.94x10^-19^ | *PDLIM1* |
| cg14219693 | 10 | 96928076 | 0.510 | 0.058 | 8.03x10^-19^ |  |
| cg19476376 | 10 | 96990921 | -0.138 | 0.016 | 1.64x10^-18^ |  |
| cg18130464 | 10 | 96039752 | -0.291 | 0.034 | 4.42x10^-18^ | *PLCE1* |
| cg04539301 | 10 | 96990923 | -0.159 | 0.018 | 4.99x10^-18^ |  |
| cg15233961 | 10 | 96990543 | -0.171 | 0.021 | 4.87x10^-16^ |  |
| cg20276630 | 10 | 97055439 | 0.313 | 0.040 | 4.89x10^-15^ |  |
| cg18389639 | 10 | 97049610 | -0.442 | 0.057 | 6.18x10^-15^ | *PDLIM1* |
| cg11911874 | 10 | 97051104 | -0.341 | 0.044 | 1.31x10^-14^ | *PDLIM1* |
| cg03178678 | 10 | 97051225 | -0.257 | 0.033 | 1.45x10^-14^ | *PDLIM1* |
| cg07347315 | 10 | 97003301 | 0.168 | 0.022 | 2.16x10^-14^ | *PDLIM1* |
| cg05599883 | 10 | 97051319 | -0.230 | 0.031 | 1.69x10^-13^ | *PDLIM1* |
| cg06570967 | 10 | 96989650 | -0.225 | 0.036 | 4.69x10^-10^ |  |
| cg04125153 | 10 | 95987486 | -0.382 | 0.061 | 4.93x10^-10^ | *PLCE1* |
| cg05771722 | 10 | 95766961 | -0.275 | 0.045 | 7.94x10^-10^ | *PLCE1* |
| cg04508033 | 10 | 96047603 | 0.204 | 0.033 | 9.05x10^-10^ | *PLCE1* |
| cg00087741 | 10 | 96961488 | -0.307 | 0.052 | 3.40x10^-09^ | *ACSM6* |
| cg13435317 | 10 | 95826508 | -0.118 | 0.020 | 7.72x10^-09^ | *PLCE1* |
| cg15776783 | 10 | 96974685 | -0.207 | 0.036 | 9.24x10^-09^ | *ACSM6* |
| cg25841553 | 10 | 96356520 | -0.102 | 0.018 | 1.94x10^-08^ | *HELLS* |
| cg10164249 | 10 | 96121853 | 0.086 | 0.016 | 4.64x10^-08^ | *NOC3L* |
| cg23153757 | 12 | 33048710 | -0.154 | 0.028 | 6.53x10^-08^ | *PKP2* |

CHR: chromosome; BP: base pair; SE: standard error
